# New-Generation Ferroelectric AlScN Materials

**DOI:** 10.1007/s40820-024-01441-1

**Published:** 2024-06-25

**Authors:** Yalong Zhang, Qiuxiang Zhu, Bobo Tian, Chungang Duan

**Affiliations:** 1 Key Laboratory of Polar Materials and Devices, Ministry of Education, Shanghai Center of Brain-Inspired Intelligent Materials and Devices, Department of Electronics, East China Normal University, Shanghai, 200241 People’s Republic of China; 2 Collaborative Innovation Center of Extreme Optics, Shanxi University, Taiyuan, 030006 Shanxi People’s Republic of China

**Keywords:** AlScN, Ferroelectrics, Nonvolatile memory, In-memory computing

## Abstract

Ferroelectrics have great potential in the field of nonvolatile memory due to programmable polarization states by external electric field in nonvolatile manner. However, complementary metal oxide semiconductor compatibility and uniformity of ferroelectric performance after size scaling have always been two thorny issues hindering practical application of ferroelectric memory devices. The emerging ferroelectricity of wurtzite structure nitride offers opportunities to circumvent the dilemma. This review covers the mechanism of ferroelectricity and domain dynamics in ferroelectric AlScN films. The performance optimization of AlScN films grown by different techniques is summarized and their applications for memories and emerging in-memory computing are illustrated. Finally, the challenges and perspectives regarding the commercial avenue of ferroelectric AlScN are discussed.

## Highlights

- Ferroelectricity and domain dynamics of emerging ferroelectric AlScN films were discussed.
- The performance optimization of ferroelectric AlScN films grown by different deposition techniques was comprehensively analyzed.
- The challenges and perspectives regarding the commercial avenue of AlScN-based memories and in-memory computing applications were summarized.

## Graphical abstract

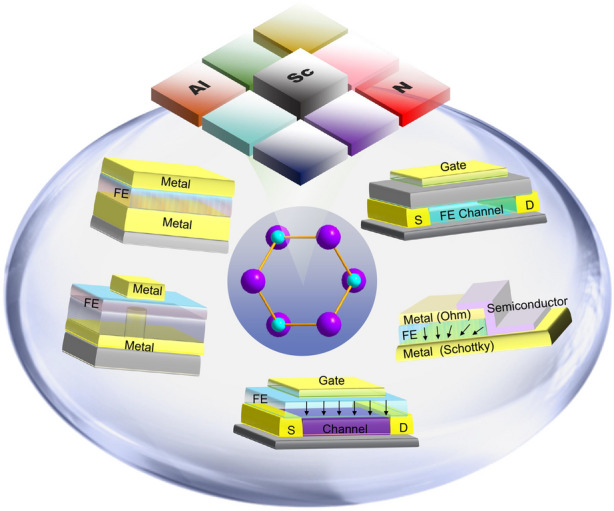

## Introduction

**Fig. 1:**
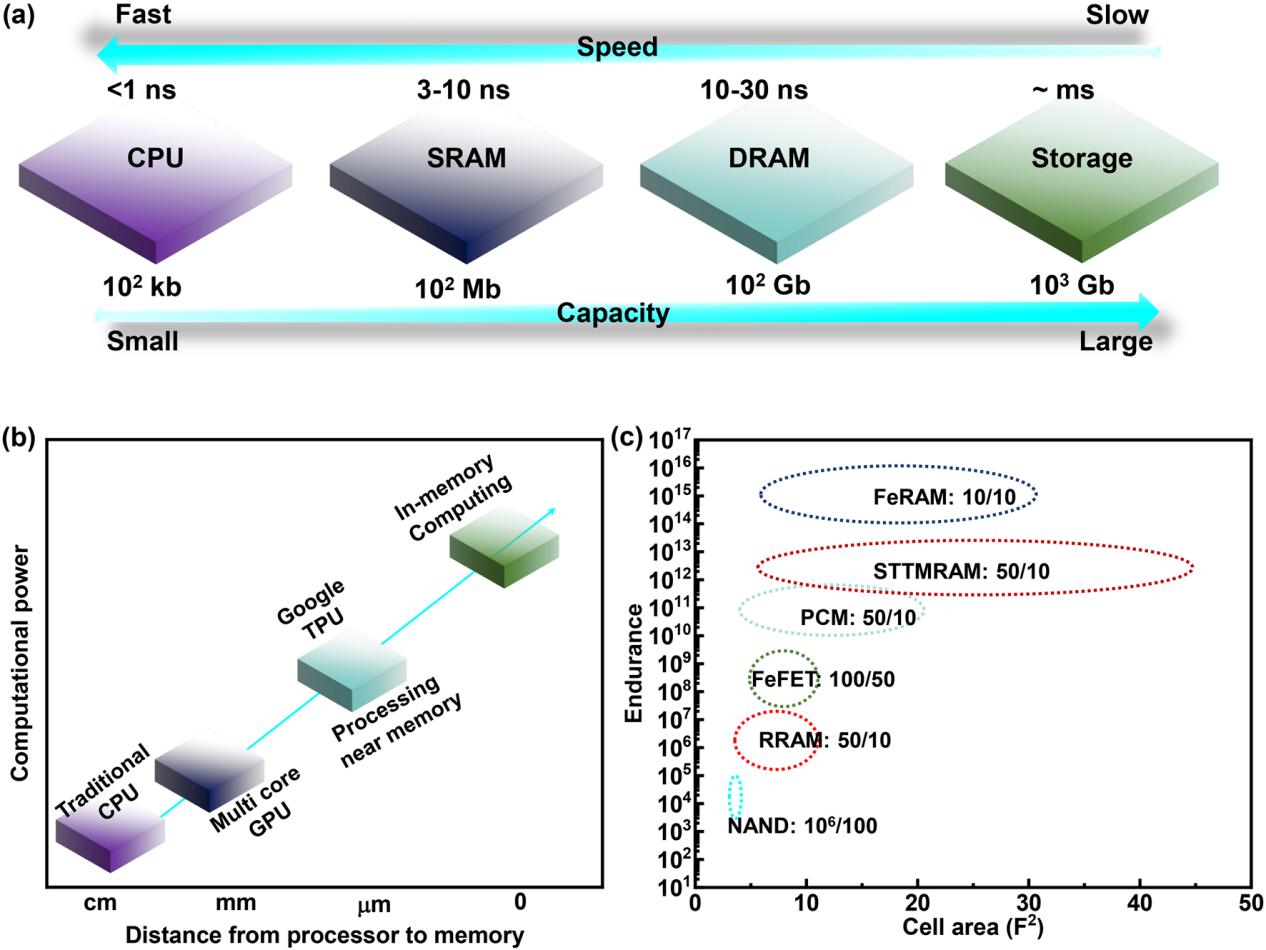
**a** Memory and CPU in von Neumann architecture. **b** The technical roadmap to improving computing efficiency. **c** The performance comparison of existing NVM. Here, “FeRAM:10/10” means that the read/write time of FeRAM is 10/10 ns, and the remaining definitions follow similar rules. Data are obtained from Ref. [19–24]

In the era of big data, artificial intelligence (AI) has made breakthroughs in the application of facial recognition, driverless driving, intelligent robots and other fields. At present, the implementation of AI is mainly based on algorithms, which require chips with large computational power for data processing. The computational power of the chip and the development of AI are complementary to each other [1–5]. The separation of the central processing unit (CPU) and memory in traditional von Neumann architecture causes latency and energy consumption during data transfer (Fig. 1a) [6, 7]. Furthermore, although CPU performance (ns level processing) has been greatly improved with the development of integrated circuit technology, the low access speed (μs level) of memory leads to severe time consumption and limits the whole performance [8–11]. In order to break through these bottlenecks, NVIDIA's multi-core graphic processing unit (GPU) and Google's tensor processing unit (TPU) with a processing near memory architecture, and in-memory computing (IMC) technology based on nonvolatile memory (NVM) have emerged in recent years (Fig. 1b) [12, 13]. In-memory computing within artificial neural networks enables highly efficient data-intensive computation due to the elimination of data migration and access. The vector–matrix multiplication (VMM) is a key operation in artificial neural networks. The crossbar array constructed with NVMs can perform VMM operation in one step following circuit laws [14]. The programmable conductance matrix is multiplied by the inputing voltage vector applied at the input wordlines in parallel to obtain current based on Ohm’s law, and the accumulated current at each bitline obeys Kirchhoff’s current law. Thanks to the science and technology advancement, a large number of NVMs emerge, including NAND flash, resistive random-access memory (RRAM), magneto-resistive RAM (MRAM), phase change RAM (PCRAM) and ferroelectric memory (FeM) [15–18]. Among them, FeM devices have unique superiorities with respect to power consumption, operation speed and endurance (Fig. 1c). For example, ferroelectric RAM (FeRAM) has faster read/write speeds and better endurance than other RAMs, and the read/write speed and endurance of ferroelectric field effect transistors (FeFETs) are also better than commercially available NAND flash. However, as will be discussed below, there is still space for the cell size of FeMs to shrink, thereby facilitating higher integration density.

Ferroelectric materials have spontaneous polarization that is switchable by electric field. Notably, multiple stable polarization states can be configurated by precisely controlling the parameters of electric field (e.g., amplitude, frequency and duration) [25–28]. It should be noted that the ferroelectric polarization states are regulated by the electric field, which avoids joule heating caused by current and significantly reduces energy consumption. The fast speed and low energy cost of polarization switching allow a high computational power of FeMs. For instance, the ferroelectric tunnel junction (FTJ) array has been reported to reach 100 tera-operations per second per watt [29]. An ideal FeM demands the involved ferroelectric materials to possess the following characteristics: (1) Good CMOS compatibility [30–32]. (2) Suitable remanent polarization (*P*_r_) to achieve more bits memory [33, 34]. (3) Optimized coercive electric field (*E*_c_) for long-term retention and large memory window, as well as excellent endurance and low operation voltage [35–37]. (4) Stable ferroelectric phase ensures the high device-to-device uniformity. Few ferroelectric materials except wurtzite-structured nitrides (e.g., AlScN, AlBN, GaScN) [38] and oxides (e.g., ZnMgO) [39], meet all above requirements simultaneously. Ferroelectricity discovered in AlScN films since 2019 brings new prosperity into FeM [40].

**Fig. 2:**
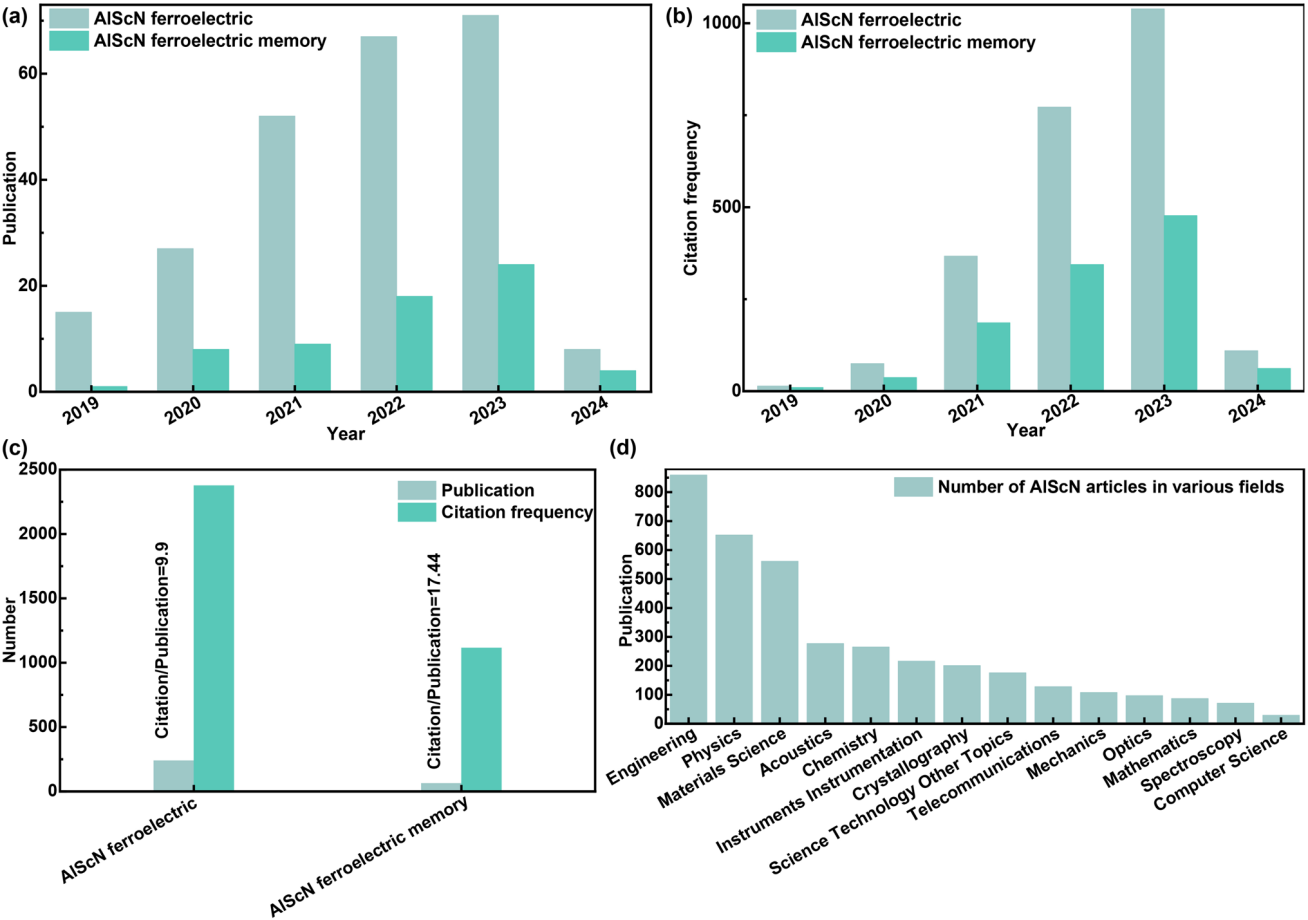
Retrieved data from Web of Science. **a** Publication, **b** citation frequency and **c** citation frequency/publication ratio of AlScN, AlScN ferroelectricity and AlScN ferroelectric memory. **d** The publication of AlScN in various research areas. Searching keywords [AlScN ferroelectric: (aluminum scandium nitride/scandium aluminum nitride/Sc-doped aluminum nitride/AlScN/ScAlN) ferroelectric; AlScN ferroelectric memory: (aluminum scandium nitride/scandium aluminum nitride/Sc-doped aluminum nitride/AlScN/ScAlN) ferroelectric memory]

Despite the fact that the number of articles about AlScN-based FeM has increased significantly with an average number of citations per article up to 17.44 since the first report of AlScN-based ferroelectric in 2019 (Fig. 2), the review article on the topic of AlScN-based FeM is rare. Therefore, it is necessary to systematically review the conspicuous and booming progresses of AlScN-based FeM. This review summarizes the latest advances in AlScN-based FeM. Chapter 2 reviews the development history of ferroelectrics and FeM. In chapter 3, the ferroelectric mechanism and domain dynamics of AlScN are discussed, and the performance optimization of AlScN thin films by various deposition methods is summarized. Chapter 4 provides an overview of AlScN-based FeM and its application in the field of IMC. In chapter 5, the challenges and perspectives of ferroelectric AlScN are discussed. This review will play a role of guiding the future development of AlScN-based memory and neuromorphic devices.

## History of Ferroelectric Materials and FeM

**Fig. 3:**
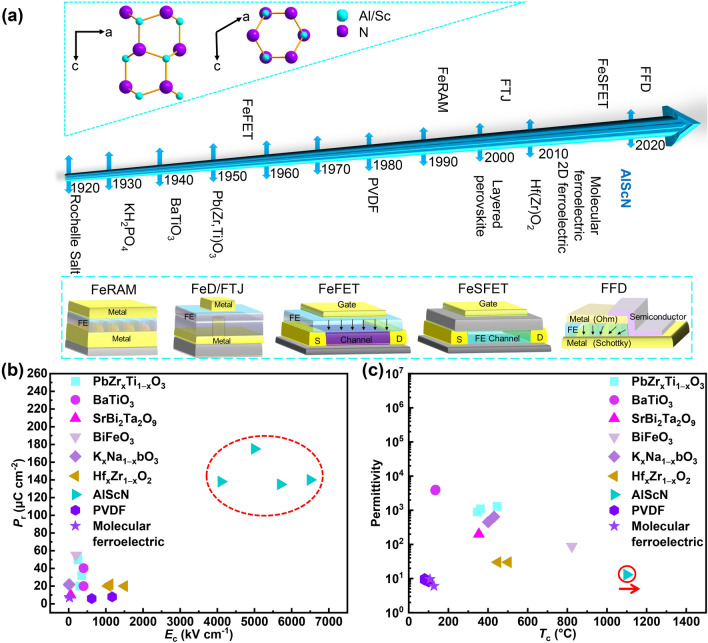
**a** Development history of ferroelectric materials and FeM. The top illustration shows the crystal structure of AlScN, and the bottom illustration exhibits the structure of FeM, wherein FeSFET, FeD and FFD represent ferroelectric–semiconductor FET, ferroelectric diode and ferroelectric fin diode, respectively. **b** Comparison of *P*_r_ and *E*_c_ between AlScN and other common ferroelectrics. **c** Comparison of permittivity and *T*_c_ among different ferroelectrics. PbZr_*x*_Ti_1−*x*_O_3_ [41–46], BaTiO_3_ [47–49], SrBi_2_Ta_2_O_9_ [50], BiFeO_3_ [51–53], K_*x*_Na_1−*x*_NbO_3_ [54, 55], Hf_*x*_Zr_1−*x*_O_2_ [56–58], AlScN [59–62], PVDF [63–66], molecular ferroelectric [67, 68]

It has been 104 years since ferroelectricity was first discovered in Rochelle salt in 1920 (Fig. 3a) [69]. Essentially, Rochelle salt was the first molecular ferroelectric crystal, but the concept of molecular ferroelectrics was proposed by Xiong et al. in the 2010s [70]. KH_2_PO_4_ was found to be ferroelectric in 1933, but it was soluble in water like Rochelle salt [71]. It was not until the 1940s that the emergence of BaTiO_3_ (BTO) and PbZr_*x*_Ti_1−*x*_O_3_ (PZT) provided the basis for the research of FeM [22, 72, 73]. In 1980, ferroelectricity was discovered in the copolymer of vinylidene fluoride and trifluoroethylene (P(VDF-TrFE)), and subsequently, poly(vinylidene fluoride) (PVDF)-based ternary and quaternary copolymers were derived. Ferroelectric polymers are widely used in wearable devices due to their good flexibility [74]. The concept of FeM was first proposed in 1952 [75], and the first commercial FeRAM based on PZT was manufactured in 1990 [76]. Immediately afterward, RAMTRON (1993), Samsung (1996) and Panasonic released their FeRAM products. However, perovskite-type ferroelectric materials are incompatible with CMOS back-end-of-line (BEOL) [77], and their performance seriously deteriorates at 130 nm or smaller process node [78, 79]. SrBi_2_Ta_2_O_9_ was used as a substitute of PZT to prepare FeRAM and its FeFETs was demonstrated, but weak oxygen binding made its performance slowly fade away [50].

Compared with the commercial development of FeRAM in the business community, other FeMs are still undergoing laboratory research. The concept of FTJ was first proposed in 1971 [80], but the requirement for high-quality ultra-thin ferroelectric films in FTJ prevented its realization until three decades later [81]. Its nondestructive conductance read mode and simple structure of FTJ are appealing for high-density memory and IMC applications. On the other hand, the poor endurance issue (usually < 10^4^) due to the ultra-thin thickness for direct quantum tunneling hinders its commercialization. The following ferroelectric diode (FD) and new-emerging ferroelectric fin diode (FFD) provide avenues to overcome the direct-tunneling limitation of FTJ memory [27]. In FFD, a ferroelectric capacitor and a fin-like semiconductor channel are combined to share both top and bottom electrodes. The intended Schottky barrier at one of semiconductor–electrode interfaces renders lateral field on the vertical semiconductor channel, resulting in ferroelectric domains-dominated resistive switching. The thickness of the ferroelectric defined by channel length does not suffer the direct quantum tunneling limit, avoiding the endurance issue in FTJ.

The prototype of FeFETs was initially proposed in the mid-1950s, which utilizes ferroelectric polarization to regulate the conductance of the semiconductor channel [82]. In 1974, Sugibuchi et al*.* prepared a metal–ferroelectric–semiconductor (MFS)-structured FeFETs, employing bismuth titanate as the ferroelectric layer and silicon as the channel [83]. It is noteworthy that the charge injection from silicon into the ferroelectric layer impacts the device performance [84]. To address this issue, Kijima et al. introduced SiO_2_ between Si and ferroelectric layer, creating a metal–ferroelectric–insulator–semiconductor (MFIS) structure [85]. However, SiO_2_, with low permittivity, tends to dissipate more voltage and cause breakdown, prompting the substitution of SiO_2_ with HfO_2_, which has higher permittivity [86]. Nevertheless, the poor interface quality of ferroelectric materials due to lattice mismatch between HfO_2_ and perovskite ferroelectric layers remains significant challenge. The emergence of two-dimensional (2D) ferroelectric semiconductors has led to the proposal of a ferroelectric–semiconductor field-effect transistor memory (FeSFET), aimed at addressing the interface issue [87].

In 2011, silicon-doped HfO_2_ was demonstrated to be ferroelectric [88]. HfO_2_-based ferroelectrics can have high *E*_c_ and *P*_r_ at ultra-thin scale (below 5 nm) and is compatible with CMOS process [88, 89]. Immediately afterward, HfO_2_-based FeFETs [90], FTJ [91] and FeRAM [92] were demonstrated one after another. In 2023, Yang et al*.* demonstrated a 9-Mb HZO-based FeRAM with 10^12^ cycle endurance [93]. However, the formation of metastable ferroelectric phase in HfO_2_-based materials requires additional post-annealing treatment, tensile stress and the presence of oxygen vacancies [94, 95]. The competition among different crystalline phases always leads to polymorphisms [96, 97], which leads to uneven performance over small area of thin film [98], and poses a problem for massively integrated circuits. Therefore, there is an urgent need for alternative ferroelectric materials.

**Fig. 4:**
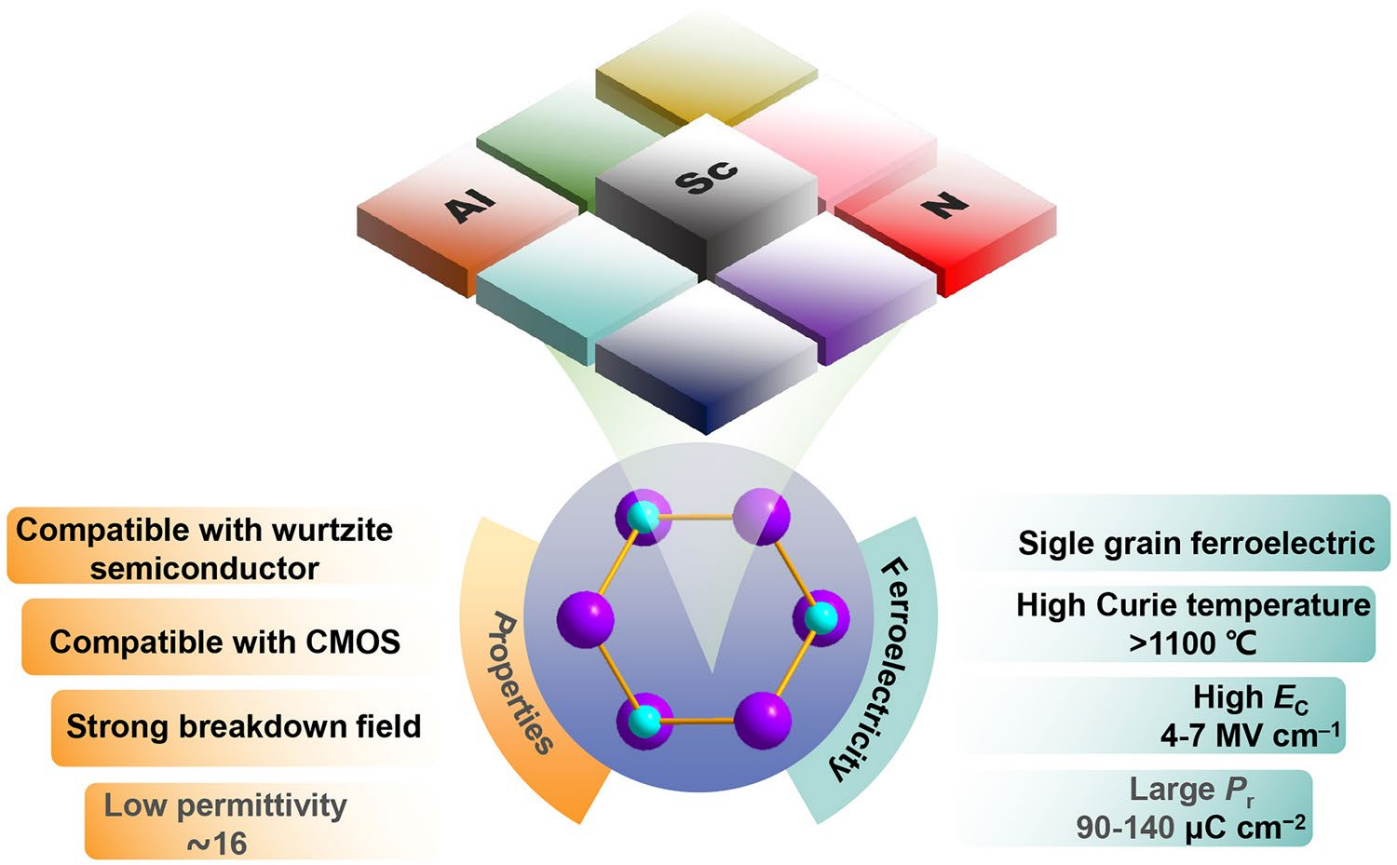
Physical properties of ferroelectric AlScN

In 2019, Simon et al*.* demonstrated ferroelectricity in AlScN [99], and its polar wurtzite phase had the lowest thermodynamic energy [100], ensuring uniform ferroelectric performance at nanoscales. It is worth mentioning that AlScN has several times larger *P*_r_ and *E*_c_ value compared with traditional ferroelectric materials (Fig. 3b). The *P*_r_ and *E*_c_ of 10 nm AlScN film reached 100 μC cm^−2^ and 11.1 MV cm^‒1^, respectively [101]. The ferroelectricity of AlScN remains stable at 1100 °C [61], making it potential for applications in aerospace and other high-temperature environments (Fig. 3c) [102]. In addition, AlScN has the lowest permittivity among known inorganic ferroelectric materials [103]. A low-permittivity ferroelectric layer can reduce the voltage sharing of non-ferroelectric layers, beneficial for increasing the sensing margin of FeRAM. In addition, ultra-thin AlScN film with thickness of 5 nm still has ferroelectricity [104, 105]. Schönweger et al*.* reported that the switching voltage of sub-5 nm AlScN was reduced to 1 V [106], enabling AlScN-based ferroelectric NVM devices to be operated by low voltage [107]. AlScN can be grown using magnetron sputtering technology below 400 °C, ensuring compatibility with CMOS manufacturing processes. The lack of volatile elements in AlScN mitigates the risk of any detrimental contamination during the COMS process [108]. In summary, AlScN is the first ferroelectric material with all following merits of stable-phase ferroelectricity, CMOS BEOL compatibility, third-generation semiconductor compatibility and sustainable scaling, etc., and has broad prospects for commercial applications (Fig. 4) [109].

## Ferroelectricity of AlScN

In order to understand the ferroelectricity of AlScN, this section will start with the crystal structures of ScN and AlN, and then introduce the relationship between ScN and AlN. Next, it will introduce the origin of AlScN's ferroelectricity, the influence of in-plane stress on AlScN's ferroelectricity due to the Sc doping, the temperature and film thickness dependence of AlScN's ferroelectricity, and the domain switching dynamics of AlScN.

### Origin of Ferroelectricity of AlScN

In common sense, ScN has stable nonpolar rock salt structure, which is difficult to connect with wurtzite AlN. However, Farrer et al*.* predicted existence of metastable hexagonal ScN with nearly five-times coordination through local-density approximation (LDA) calculations [110]. Subsequently, Ranjan et al*.* conducted LDA calculations to predict the structural phase transition of hexagonal ScN from nonpolar to polar under continuous compressive strain [111]. The above prediction of hexagonal ScN provides a prerequisite for the birth of ferroelectric Sc–Al–N in the future.

AlN with wurtzite structure (space group *P*6_3_*mc*) has polarization along [001] direction, arising from the separation of Al^3+^ cation and N^3‒^ anion centers [112, 113]. Therefore, there are two antiparallel polarization directions: N-polar and Al-polar [114]. Pure AlN is piezoelectric rather than ferroelectric material, because polarization cannot be switched by an electric field lower than dielectric breakdown limit [115, 116]. In other words, the polarization of wurtzite AlN would be switchable if reducing the energy barrier between the two polarization states.

Owing to the ultra-high thermal stability of wurtzite AlN, it is difficult to directly study the phase transition process as a function of temperature [60]. However, the pressure-induced phase transition process in AlN will bring some new ideas [117]. Vollstädt et al*.* demonstrated the phase transition from wurtzite to rock salt structure at 14–22 GPa [118]. Zagorac et al*.* predicted the first-order phase transition of AlN from wurtzite to rock salt at a pressure of 19 GPa [119]. Regarding the phase transition path of AlN from wurtzite to rock salt, most scholars support the view that layered hexagonal phase serving as an intermediate phase is energetically favorable [120, 121].

**Fig. 5:**
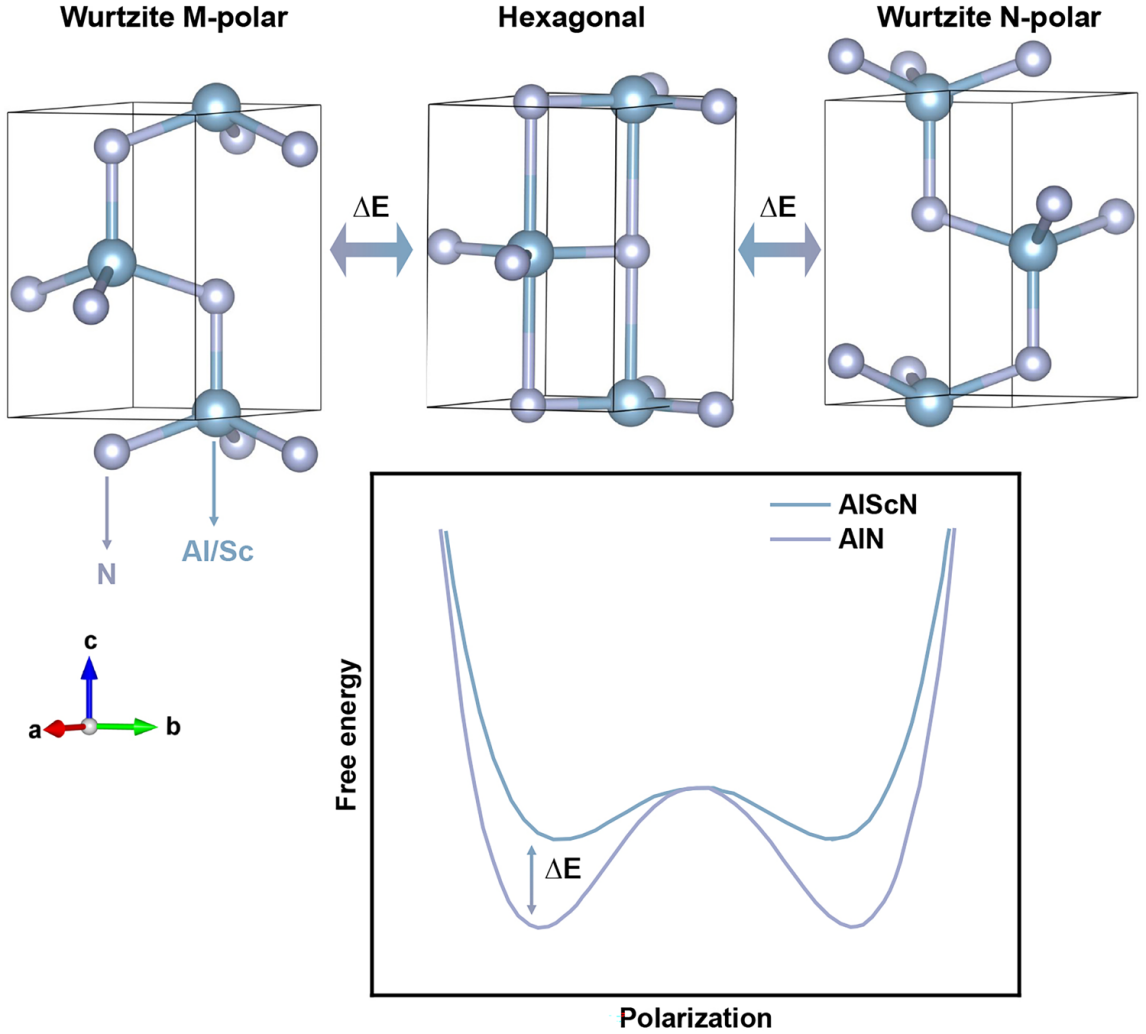
Polarization switching process of AlScN and the change of double-well potential after Sc doping

Tasnádi et al*.* revealed that Sc doping can flatten the energy landscape of AlN [122]. Wang et al*.* predicted that the ferroelectric switching barrier of AlScN decreased with the increase in Sc content, enabling the switching between Al-polar and N-polar in AlScN [123]. Zhang et al*.* speculated that AlScN shows stable polar wurtzite phase when Sc content is lower than 0.56, and nonpolar rock salt phase when Sc content is higher than 0.56 [116]. The ferroelectricity of AlScN is suggested to be related to the existence of metastable layered hexagonal phase of ScN (space group *P*6_3_/*mmc*) [99], which plays the role of the transition state between the two polarization orientations of wurtzite structure to reduce the energy barrier between two polarization states (Fig. 5) [110].

### Factors Affecting the Ferroelectricity of AlScN

**Fig. 6:**
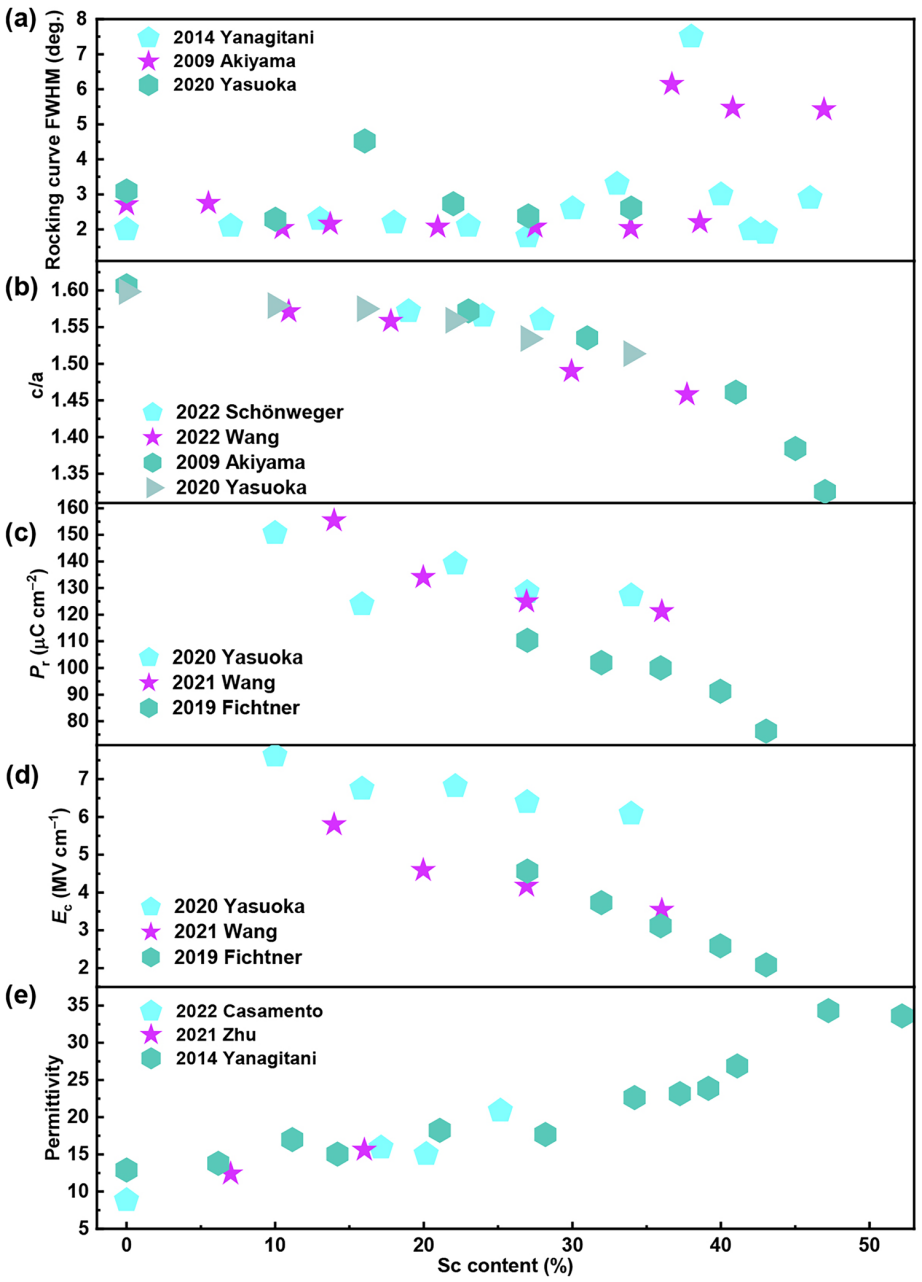
Material properties of Al_1−*x*_Sc_*x*_N as a function of the Sc concentration. **a** Rocking curve FWHM, **b** *c*/*a*, **c** *P*_r_, **d** *E*_c_ and **e** permittivity. Data from Ref. [59, 99, 103, 124–129]

Despite theoretical predictions as a guide, it is challenging to lower the polarization switching barrier while maintaining the dielectric breakdown strength of the material. Until 2019, Fichtner et al*.* demonstrated the ferroelectric polarization switching in AlScN with *P*_r_ of 110 μC cm^−2^ [99]. Immediately afterward, a large amount of research work explored the factors affecting the ferroelectricity of AlScN. In order to more objectively evaluate the impact of Sc content on the ferroelectricity of AlScN, the research data from different groups are compiled here for comparison (Fig. 6). As mentioned in Sect. 3.1, Sc doping can reduce the energy barrier between the two polarization states of Al_1−*x*_Sc_*x*_N, allowing polarization reversal under external voltage. This has been experimentally confirmed. With the increase in Sc content, the *E*_c_ of AlScN decreases (Fig. 6d), implying that the switching barrier of AlScN is indeed lowered with the incorporation of Sc. The evolution of *E*_c_ in Al_1−*x*_Sc_*x*_N with the Sc content (*x*) satisfies the following relationship: ${E}_{c}\left(x\right)=-15x+8.35$ (MV cm^‒1^) (0 < *x* < 0.43) [130]. However, when the Sc content is greater than 30%, the full width at half maximum (FWHM) of the rocking curve for (0002) diffraction peak shows a step increase, indicating that crystallization deterioration is observed (Fig. 6a). The reduction of the switching barrier of AlScN through the incorporation of Sc is generally regarded to relate with the in-plane tensile strain generated by the structural distortion of the wurtzite crystal, which can be confirmed by the decrement of *c*/*a* values in Fig. 6b [131]. As the Sc content further increases, Al_1−*x*_Sc_*x*_N gradually transforms from the ferroelectric wurtzite phase to the non-ferroelectric rock salt phase, and the *P*_r_ value rapidly decays (Fig. 6c). In-plane mechanical stress on AlScN film yielded similar impacts. With the in-plane mechanical stress changes from − 0.8 to + 0.5 GPa, *E*_c_ decreases from 5 to 4 MV cm^−1^ [99]. Both Sc doping and tensile stress have a tendency for fivefold coordination in a ScN-like planar hexagonal structure, leading to a sufficient energetic destabilization of the wurtzite structure to allow for ferroelectric switching. In addition, the permittivity of Al_1−*x*_Sc_*x*_N slightly increases with the increase in Sc content (Fig. 6e). To obtain better ferroelectric performance, Sc content and in-plane stress should be optimized for reducing the *E*_c_ and leakage current and maintaining the breakdown strength [100, 132].

**Fig. 7:**
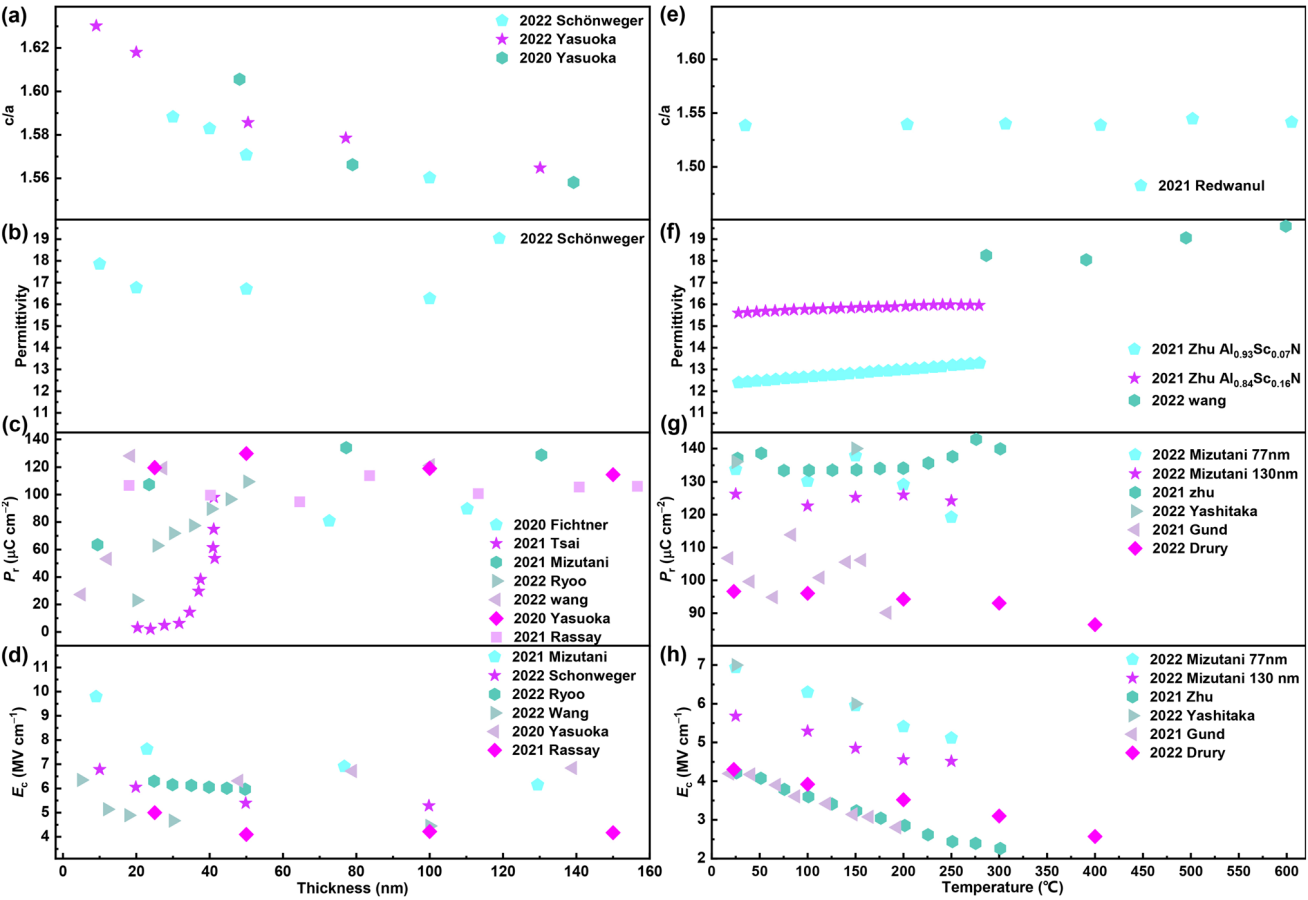
Material properties of Al_1−*x*_Sc_*x*_N as a function of the film thickness. **a** *c/a*, **b** permittivity, **c** *Pr* and **d** *Ec*. The material properties of Al_1−*x*_Sc_*x*_N as a function of temperature. **e** *c/a*, **f** permittivity, **g** *Pr* and **h** *Ec*. Data from Ref. [99, 101, 103, 104, 126, 127, 133–139]

The thickness dependence of ferroelectricity of Al_1−*x*_Sc_*x*_N films was comprehensively explored. For the case of in-plane tensile strain generated by the substrate, the strain will relax with the increase in film thickness. Correspondingly, the *c*/*a* of Al_1−*x*_Sc_*x*_N decreases with the increase in film thickness within 50 nm and tends to be stable for film thickness greater than 50 nm (Fig. 7a). The dielectric constant of Al_1−*x*_Sc_*x*_N is slightly affected by the film thickness (Fig. 7b). As the film thickness decreases, the measured *P*_r_ value of Al_1−*x*_Sc_*x*_N decreases. This decay phenomenon of polarization in thinner films is mostly related to the dead layer at interface (Fig. 7c). A good epitaxial quality can endow a high *P*_r_ value of 100 μC cm^−2^ at 100 °C for a 9-nm Al_1−*x*_Sc_*x*_N films [101]. Following a nucleation dynamics, the scaling of the *E*_c_ with thickness in traditional ferroelectric films obeys ${E}_{c}\propto {d}^{-2/3}$, where *d* is thickness of the ferroelectric films [140]. In Al_1−*x*_Sc_*x*_N thin films, as the thickness decreases, the *E*_c_ remains relatively stable and begins to increase when the film thickness reaches below 20 nm (Fig. 7d) [141].

Vast reports reveal that Al_1−*x*_Sc_*x*_N exhibits stable temperature dependence. The *c*/*a* of Al_1−*x*_Sc_*x*_N slightly change with temperature (Fig. 7e) [60]. As the temperature increases, the permittivity of Al_1−*x*_Sc_*x*_N also changes very little, less than 5% within 600 °C (Fig. 7f). Within 400 °C, the *P*_r_ of Al_1−*x*_Sc_*x*_N shows good temperature stability (Fig. 7g). In addition, the *E*_c_ of Al_1−*x*_Sc_*x*_N decreases with the increase in temperature. From room temperature to 300 °C, the *E*_c_ of Al_1−*x*_Sc_*x*_N decreases by half (Fig. 7h). However, compared with other ferroelectric materials, this *E*_c_ value is still too large.

### Polarization Switching in Ferroelectric AlScN

**Fig. 8:**
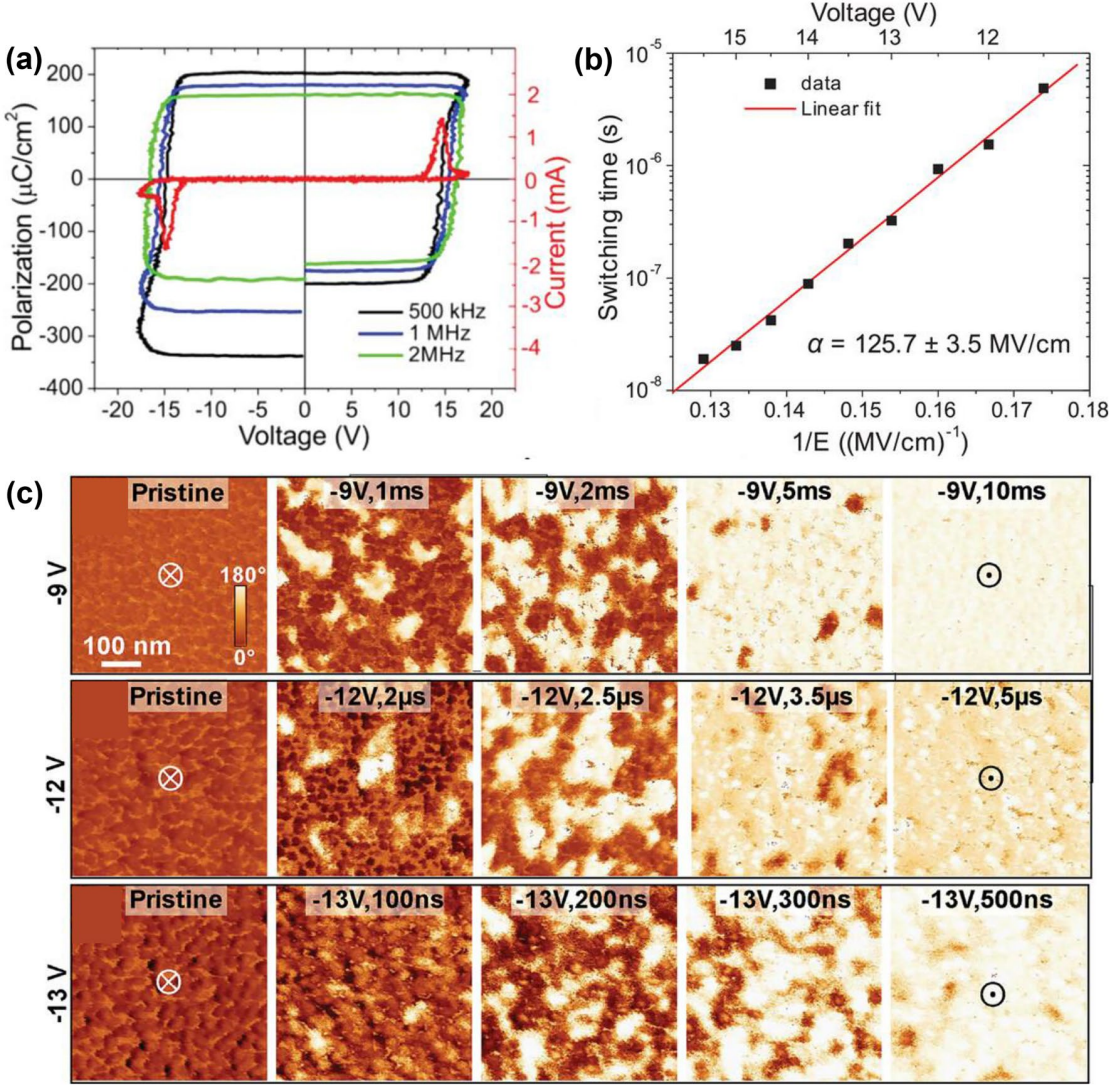
**a** *P*–*V* loops of Al_0.72_Sc_0.28_N at various frequencies. **b** Switching time as a function of the pulse amplitude. **c** PFM phase images of Al_0.72_Sc_0.28_N after exerting voltage pulse with amplitude of ‒9 V, ‒12 V and ‒13 V. Reproduced with permission from Ref. [142]. Copyright 2024 Wiley-VCH GmbH

**Fig. 9:**
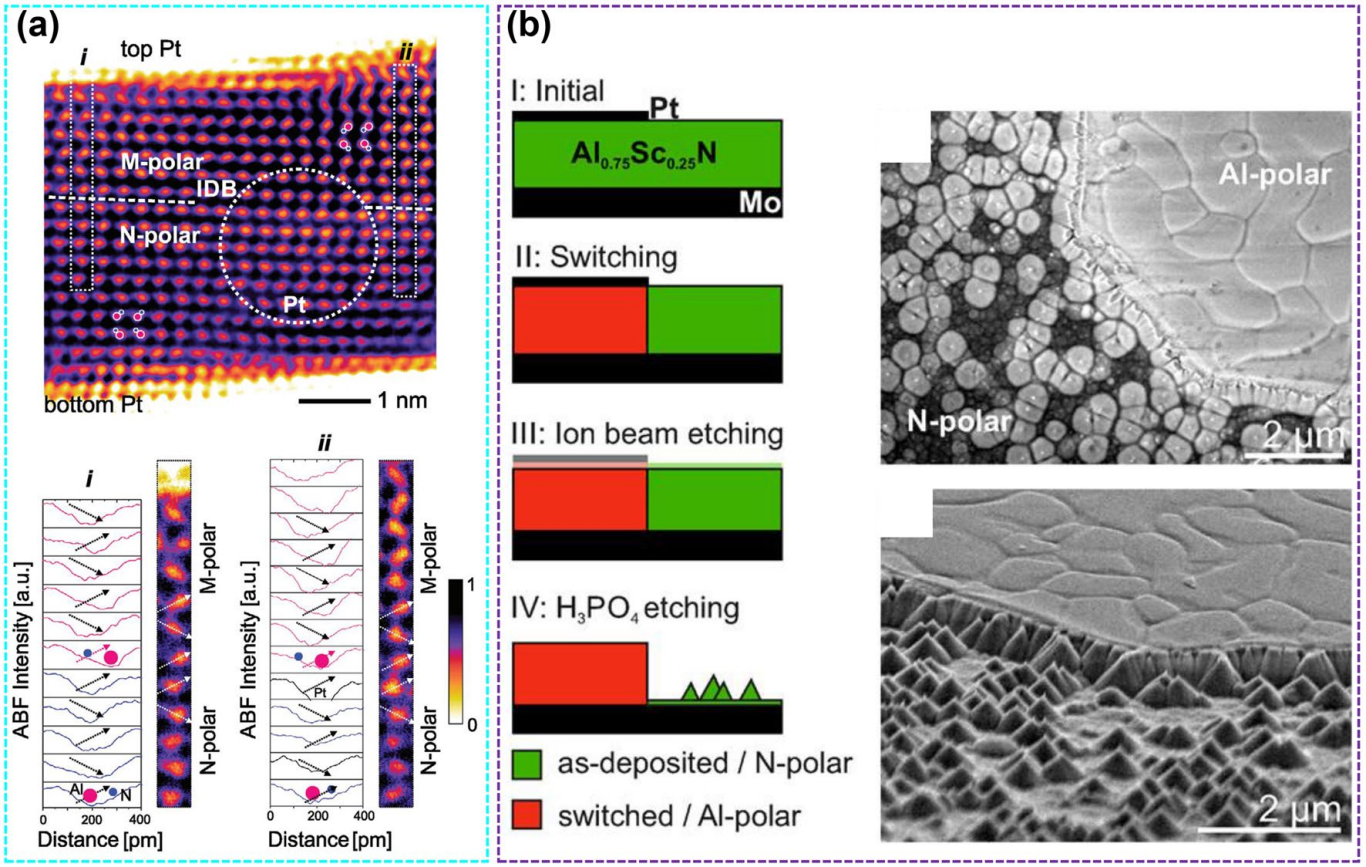
**a** STEM micrographs of the M- and N-polar domain boundaries of Al_0.74_Sc_0.26_N. Reproduced with permission from Ref. [106]. Copyright 2023 Wiley-VCH. **b** Schematic diagram of determining local metal or N-polar by H_3_PO_4_ etching method. Reproduced with permission from Ref. [148]. Copyright 2021 AIP Publishing

It is important to explore the polarization switching mechanism of ferroelectric AlScN. The regions with uniform spontaneous polarization in ferroelectrics are called domains, and the boundaries between domains with different polarization directions are called domain walls. Generally, according to whether the angle between the spontaneous polarization directions of two adjacent domains is 180°, they are divided into 180° domain walls and non-180° domain walls [143]. Common non-180° domain walls include 90° domain walls, represented by the tetragonal and orthorhombic phases, and 71° and 109° domain walls represented by the rhombohedral phase [144]. The polarization switching process of ferroelectrics includes domain nucleation and growth. Lu et al. used piezoresponse force microscopy (PFM) that combines a pulse testing to reveal the evolution of the domain structure of Al_0.72_Sc_0.28_N capacitors over time under different voltages [142]. Al_0.72_Sc_0.28_N capacitors have *P*_r_ exceeding 150 μC cm^−2^, and its hysteresis loop has a steep slope (Fig. 8a). With the increase in voltage (from 11.5 to 15.5 V), the switching time of Al_0.72_Sc_0.28_N capacitor decreases by three orders of magnitude, which is consistent with the changes in domain structure displayed by PFM over time at different voltages (Fig. 8b, c). Zhang et al*.* observed the formation of ferroelectric domains in AlN that has some Sc content, through 4D-STEM differential phase contrast mapping [145]. The same characterization method also revealed the distribution of ferroelectric domains in single-crystal AlScN nanowires [146]. Kim et al*.* confirmed that the domain nucleation growth mechanism of AlScN satisfies the inhomogeneous field model (IFM) [147]. The characteristic switching time limit of AlScN (5.98 × 10^−14^ s) is more than three orders of magnitude faster than that of HZO (1 × 10^−10^ s), while its activation field (96 MV cm^−1^) is one order of magnitude higher than that of HZO (8.94 MV cm^−1^), accounting for the high *E*_c_ of AlScN. Schönweger et al. firstly reported inversion domain boundaries in single crystal of AlScN, which supports the domain reversal theory of gradual domain-wall driven switching process (Fig. 9a) [106].

Polarization switching of AlScN on a macroscopic scale has been observed. The crystal cell orientation of wurtzite semiconductors (GaN, AlN, etc.) can be verified through acid–base etching (H_3_PO_4_, KOH, etc.) [149]. The surface of N-polar is easy to be etched by acid and alkali, with conical shape remaining on the surface, while the metal-polar surface is almost not etched (except for local defects and reverse domains) [150]. Niklas et al. confirmed the conversion of N-polar to metal-polar after ferroelectric polarization by H_3_PO_4_ etching method. The corresponding scanning electron microscope (SEM) shows the conical morphology of the N-polar surface after etching and transition from N-polar surface to metal-polar surface after polarization reversal (Fig. 9b).

At the atomic scale, when ferroelectrics undergo polarization switching under the action of an electric field, it is often accompanied by the relative displacement of anions and cations [152]. For example, BaTiO_3_ is the displacement of Ba^2+^ cations relative to Ti–O octahedron, while in HfO_2_-based ferroelectrics it is the displacement of O^2−^ anions relative to metal cations [141]. As we know, the polarity of AlN is derived from the relative separation of Al^3+^ and N^3‒^ anions. Combined with the previous experience of traditional ferroelectrics, the polarization switching of AlScN under the action of an electric field is also accompanied by the relative displacement of metal cations and N^3‒^ anions.

**Fig. 10:**
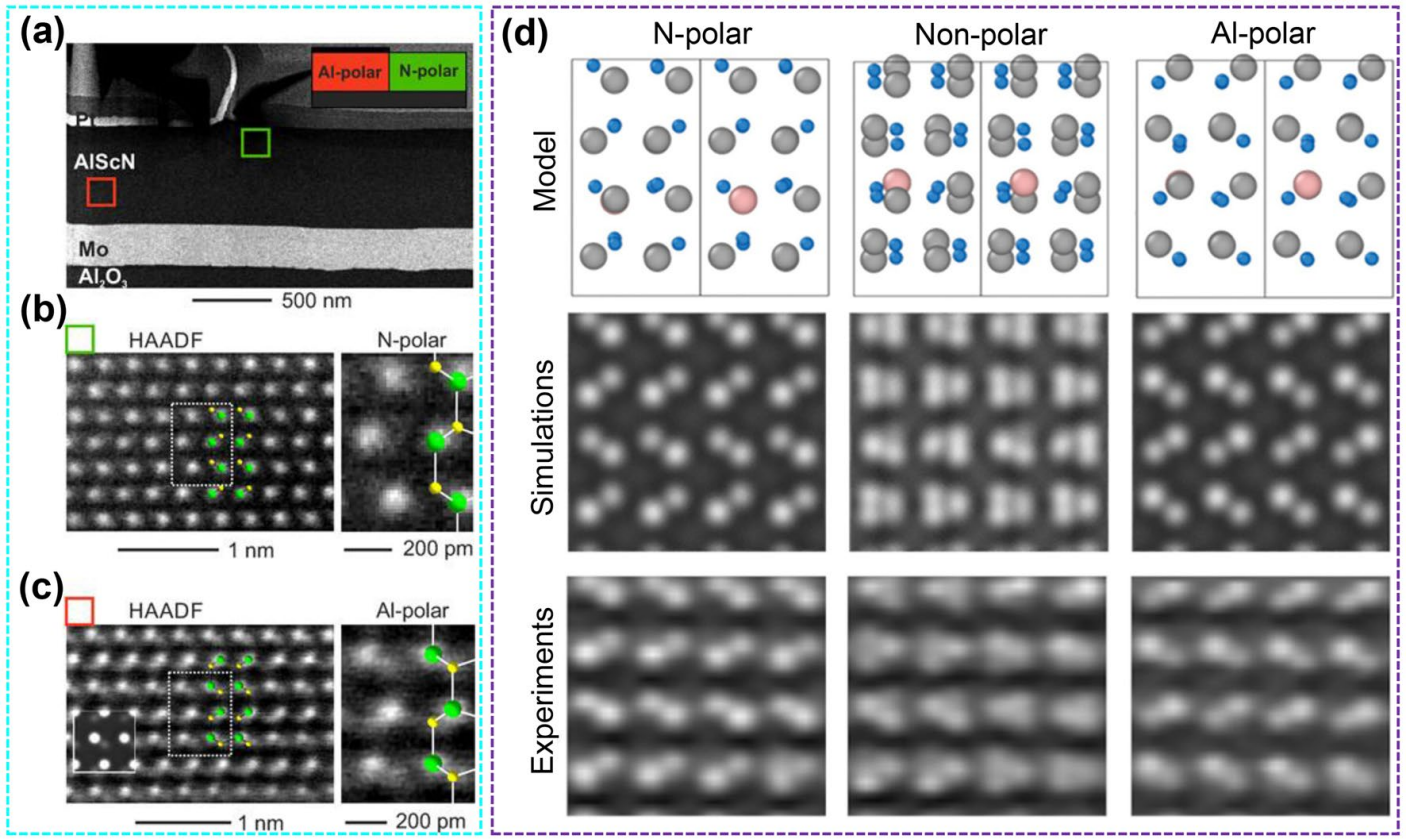
**a** STEM image of deposited Pt area with polarization switched to metal-polar and unswitched area (N-polar). **b** HAADF micrographs show the atomic structure of N-polar. **c** HAADF micrographs show the atomic structure of metal-polar after polarization reversal in the deposited Pt region. Reproduced with permission from Ref. [148]. Copyright 2021 AIP Publishing. **d** Atomic models, STEM image simulations and experimental images for the N-polar, nonpolar and Al-polar states. Reproduced with permission from Ref. [151]. Copyright 2023 AAAS

The transformation of N-polar to metal-polar after ferroelectric polarization is confirmed from the atomic scale by STEM. The green frame line is the initial N-polar surface and the red frame line is the metal-polar surface after inverted polarization (Fig. 10a). The images of high angle annular dark field (HAADF) in the green area show obvious N-polar (Fig. 10b), while the images of HAADF in the red area show that the unit cell polarity is reversed from N-polar to metal-polar (Fig. 10c) [148]. Sebastian et al*.* successfully observed the atomic-scale polarization switching process of AlN-based ferroelectrics during voltage application through in situ STEM, that is, from the initial (N-polar), intermediate (nonpolar) and final (Al-polar) switching sequence (Fig. 10d) [151]. So, the polarization switching of ferroelectric AlScN is the mutual switching between metal-polar and N-polar under the action of an electric field, and this process is accompanied by the relative displacement of metal (Al^3+^ and Sc^3+^) cations and N^3‒^ anions under the action of an electric field.

The above insights into the macroscale and in situ atomic-scale processes of polarization switching in AlN-based ferroelectric materials provide guidance for future research on other wurtzite-structured ferroelectric materials, and it also provides a strong theoretical support for the future commercial application of AlN-based ferroelectrics.

### Performance Optimization of AlScN Films Grown by Different Deposition Techniques

It is essential to fabricate high-quality AlScN films to meet the application requirements. So far, AlScN thin films can be deposited through the various techniques, including molecular beam epitaxy (MBE), magnetron sputtering (MS), metal–organic chemical vapor deposition (MOCVD) and pulsed laser deposition (PLD). First of all, it is important to understand that both Al and Sc have high oxygen affinity, so AlScN needs to be grown in a high vacuum environment to avoid oxygen defects. Substrates, such as typically Pt (111), Mo (110), Al (110), GaN, AlN, Al_2_O_3_ and other substrates can provide a hexagonal template for AlScN.

The advantages of MS include relatively low cost, good repeatability, strong adhesion between the film and the substrate, and the ability to deposit films with different element content [153]. MS deposition of AlScN commonly uses Al/Sc alloy targets or employing co-sputtering via two metal targets. The alloy target offers a higher deposition rate, which can ensure uniform composition across the entire wafer and thus is suitable for industrial applications. However, adjusting the scandium content in the alloy target can be challenging, and the complex metallurgical processes involved may result in higher costs. Co-sputtering can tune the Sc concentration by adjusting the power of the single target, which is lower in cost. However, reducing the power of the single target can compromise the uniformity and crystallinity of the film. Parameters such as power, N_2_/Ar ratio, background pressure and growth temperature during sputtering affect the sputtering rate, film quality, surface roughness and electrical properties of AlScN. For example, the N_2_/Ar ratio governs both the sputtering rate and nitridation reaction, while the sputtering power regulates the sputtering rate and crystallinity. Background pressure impacts the sputtering rate, whereas growth temperature influences the grain size, film surface stress and crystallinity. Chiu et al. studied the effect of sputtering conditions on the crystal quality of AlN thin films [154]. They identified the optimal deposition conditions for AlN as follows: power = 1400 W, N_2_/Ar = 80%, background pressure = 0.8 Pa and growth temperature = 400 °C. A few studies have reported the impacts of different deposition conditions on the ferroelectricity of AlScN. For example, increasing the N_2_/Ar ratio leads to higher values of both *P*_r_ and *E*_c_ increase, alongside the decreased film roughness and increase the stress [155]. The AlScN film is deposited at 400 °C exhibits the highest *P*_r_ [156]. Ryoo et al*.* systematically investigated the effects of different magnetron sputtering parameters (power, N_2_/Ar ratio, background pressure and growth temperature) on the ferroelectric properties of Al_0.7_Sc_0.3_N. The exploration of depositional conditions is outlined in Table 1, and will not be further discussed here. The optimum deposition conditions of Al_0.7_Sc_0.3_N on TiN were determined as follows: power = 500 W, N_2_/Ar = 100%, background pressure = 20 mTorr and growth temperature = 300 °C. Under these deposition conditions, the maximum saturated 2 *P*_r_ of 120 μC cm^−2^ for 25-nm films was observed, which was about half of that in 50-nm-thick films (≈ 220 μC cm^−2^) [135]. The substrates with different thermal expansion coefficients also impact the ferroelectric properties of AlScN films. Due to the existence of thermal stress, the lattice parameters of the films are affected. Results demonstrate that the *P*_r_ and *E*_c_ of the AlScN film decrease with the decrease in the thermal expansion coefficient of the substrate [157]. High Sc content can lead to impurities (rock salt) formation and an increase in defect density, which can affect crystal quality and elevate leakage current. In addition, the MS technique used to deposit AlScN epitaxial films on single-crystal GaN substrates is accompanied by a competition between M-polar and N-polar, especially when AlScN exceeds 30 nm, resulting in the coexistence of multiple domains [127]. Table 2 summarizes the conditions for depositing AlScN with MS and the corresponding ferroelectric properties of AlScN.

**Table 1:** Effect of MS deposition conditions on the ferroelectric properties of AlScN. Data collected from Ref. [135]

	*c* (Å)	*a* (Å)	Lattice volume (Å^3^)	Deposition rate (nm min^−1^)	Grain diameter (nm)	Roughness (nm)	Sc/(Sc + Al)	*P*_r_ (μC cm^−2^)	*E*_c_ (MV cm^‒1^)
Power									
300 W	4.981	3.211	133.411	1.02	13.154	0.403	0.304	145.84	5.69
400 W	4.986	3.198	132.453	1.632	12.258	–	0.308	153.33	5.78
500 W	4.988	3.194	132.181	2.28	13.338	0.725	0.306	153.5	5.83
600 W	4.997	3.19	132.076	2.928	14.392	0.733	0.316	134.18	5.94
N_2_/Ar									
20%	4.99	3.218	134.248	2.97	12.418	0.593	0.308	113.45	5.62
40%	4.999	3.214	134.133	2.562	–	0.668	–	141.8	5.75
60%	4.997	3.203	133.173	2.28	14.072	0.685	0.297	153.35	5.8
80%	5.005	3.202	133.289	2.25	–	0.802	–	159.35	5.81
100%	5.017	3.192	132.768	2.1	13.832	0.992	0.300	156.2	5.92
Pressure									
5 mTorr	5.001	3.192	132.405	2.04	13.646	0.977	0.296	142.9	6
10 mTorr	5.001	3.186	131.866	1.806	12.29	0.817	0.312	161.6	6.1
15 mTorr	5.031	3.198	133.663	1.548	10.936	0.773	0.298	159.71	6.2
20 mTorr	5.064	3.203	134.995	1.35	9.136	0.717	0.302	177.83	6.37
Temperature									
RT	5.052	3.189	133.501	1.272	10.626	0.898	0.308	161.5	6.34
150°C	5.024	3.185	132.431	1.272	11.2	0.822	0.303	174.7	6.12
300°C	5.015	3.195	133.01	1.29	12	0.392	0.296	201.43	6
400°C	5.012	3.2	133.333	1.26	11.8	0.301	0.297	186.2	5.9

**Table 2:** MS deposition of AlScN

Substrate	Target	Sc composition	Growth temperature (°C)	Thickness (nm)	FWHM (°)	*P*_r_ (μC cm^−2^)	*E*_c_ (MV cm^‒1^)	References
AlN/Pt	AlSc alloy	0.27–0.43	400	400–1000	–	75–110	2–5	[99]
Pt/TiO_*X*_/SiO_2_/Si	Al and Sc	0.1–0.34	400	9–140	2–2.7	3.8–135	–	[126]
Pt/Si	Al and Sc	0.29	350	100	–	80	–	[158]
Pt(111)/Ti/SiO_2_/Si(100)	Al and Sc	0.36	–	20	2.8	30	6.5	[159]
Al_2_O_3_	Al and Sc	0.16	350	363	–	135	4.2	[103]
Pt/Si	Al and Sc	0.36	350	20	–	25	6.5	[160]
Mo/Si	Al and Sc	0.27	–	400–2000	–	138	4.1	[60]
Mo/AlN/Al_2_O_3_	Al and Sc	0.25	450	550	2.6	–	–	[148]
Al/Al_0.80_Sc_0.20_N /Si	Al and Sc	0.32	150	45	–	115	− 4.3/5.3 (10 kHz)	[161]
Pt/SiO_2_/Si or Al_2_O_3_ or MgO	Al and Sc	0.2	400	100		115–127	6.5–7.2	[157]
*n*-GaN	Al and Sc	0.19–0.28	450	40–300	0.28–0.38	≤ 120	6–12	[127]
Pt/Ta/SiO_2_	Al and Sc	0.2–0.45	400	90–200	–	–	–	[162]
Pt(111)/TiO_*X*_/SiO_2_/Si(100)	Al and Sc	0.2	400	12–130	–	~ 135	5–7	[133]
Al	Al and Sc	0.32	375	45	–	120	–	[163]
Pt/TiN	Al and Sc	0.38	RT or 400	15	–	–	–	[164]
Pt/GaN	Al and Sc	0.28	500	100	2.44	~ 175	5	[61]
Pt (111)/Ti/SiO_2_/Si	Al and Sc	0.32–0.36	350	20	2	140	6.5	[62]
TiN	Al and Sc	0.22	400 and RT	50	9.7 at 400 °C, 15.2 at RT	113 at 400 °C, 70 at RT	8.1 at 400 °C, 6.5 at RT	[130]
Pt/TiO_*X*_/SiO_2_/Si	Al and Sc	0.2	400	9–130	–	> 100	6–10	[101]
Pt/TiO_*X*_/SiO_2_/Si	Al and Sc	0.22	25–500	120–190	–	10–129	6–8	[165]
Pt/GaN	Al and Sc	0.28	450	10–100	0.1–2	–	5.5–6.5	[107]
Pt	Al_0.7_Sc_0.3_ alloy	0.3	400	225	2.694	100	4.3	[102]

The epitaxial growth of AlScN on GaN, AlN, SiC, Al_2_O_3_ and Mo substrates can be achieved through MBE, MOCVD and PLD techniques. The advantage of MBE is that ultra-thin epitaxial films with high uniformity can be prepared, and multilayer structure with different dopants or components can be realized [166]. At present, the temperature for depositing AlScN by MBE is typically above 500 °C. Wang et al*.* grew AlScN epitaxial films on GaN substrates by MBE, and confirmed that the optical bandgap of AlScN decreased with the increase in Sc content. The Sc_0.20_Al_0.80_N grown by MBE almost matches the GaN lattice, showing a *E*_c_ of ~ 4.2 MV cm^‒1^ and a *P*_r_ of ~ 135 μC cm^−2^. The polarization retention time of Sc_0.20_Al_0.80_N is more than 10^5^ s and there is no fatigue behavior with 3 × 10^5^ cycles [109]. In order to meet the requirements of low-power memory and CMOS compatibility, devices need to operate at low voltages, grow on Si substrates at low-temperature. Reducing the film thickness can reduce the operating voltage [167]. Wang et al*.* have grown 5–100-nm-thick Al_0.7_Sc_0.3_N epitaxial film on Mo/Si substrate. With the decrease in film thickness, the *E*_c_ increases while the *P*_r_ decreases. Specifically, 5 nm thick Al_0.7_Sc_0.3_N can achieve *P*_r_ of 23 μC cm^−2^ at 2–3.8 V [104]. Growth in a nitrogen-rich environment can ensure the phase purity and crystal quality of wurtzite AlScN, but can lead to increased film roughness [168]. Hardy et al. grew high-quality AlScN films at 390 °C, enabling growth of AlScN at COMS compatible process temperature [169]. It is noteworthy that the ferroelectric properties of AlScN grown by MBE are comparable to those of AlScN grown by MS. Table 3 summarizes the epitaxial deposition of AlScN and its ferroelectric properties under corresponding deposition conditions.

**Table 3:** Epitaxial deposition of AlScN

Method	Substrate	Sc composition	Growth temperature (°C)	Thickness (nm)	FWHM (°)	*P*_r_ (μC cm^−2^)	*E*_c_ (MV cm^‒1^)	References
MBE	*n*-GaN	0.17–0.25	600	100	0.03–0.05	–	–	[129]
	Si <111>	0.03–0.26	–	400–800	1.1	–	–	[170]
	Si-doped-GaN	0.2	–	200	–	70–80	4.6	[171]
	*n*-GaN	0.18–0.4	600–750	25–30	0.09–0.12	–	–	[172]
	Si	0.12	–	400	1.2	–	–	[173]
	AlN	0.06–0.32	700/390	200	–	–	–	[169]
	GaN/AlN	0–0.34	400–900	50–100	–	–	–	[174]
	Si-doped-GaN	0.14–0.36	–	100	–	135	3.4–5.7	[59]
	Si-doped-GaN	0.21	–	–	0.31	90	4.9	[175]
MOCVD	GaN/Al_2_O_3_	0.05–0.17	1000–1200	6.5–16.9	–	–	–	[176]
	GaN/Al_2_O_3_	0.2–0.3	1000	10–100	0.07	–	–	[177]
PLD	Mo/Si	0.3	–	100	–	140	5	[178]

MOCVD can control the composition and doping level of compounds by quickly switching gas source. It facilitates the growth of the single-crystal film over large area with high uniformity and repeatability, achieving a relatively high growth rate (micron h^−1^), so it stands as the preferred technology for the manufacturing nitride semiconductors (AlN, GaN) [179]. However, a significant challenge of growing AlScN by MOCVD lies in the lack of Sc precursors capable of providing sufficient vapor pressure. In 2019, Stefano et al*.* successfully deposited Al_1−*x*_Sc_x_N (*x* = 0.05–0.17) epitaxial film on GaN substrate at 1000–1200 °C using tris-cyclopentadienyl-scandium as the precursor of Sc for the first time [177]. SiN_*X*_-passivated AlScN/GaN heterostructures are used to fabricate a high-electron mobility transistor based on AlScN, demonstrating a transconductance of nearly 500 mS mm^−1^ [176]. Moreover, the purity of the Sc precursor source affects the leakage current [180]. However, there is currently no report on the ferroelectricity of AlScN grown by MOCVD. Recently, Liu et al*.* deposited 100-nm-thick Al_0.7_Sc_0.3_N thin film on Mo substrate by PLD, with *P*_r_ of 140 μC cm^−2^ and *E*_c_ of 5 MV cm^‒1^ [181]. The advantage of PLD is its capability to obtain multi-component films with desired stoichiometry, but it is not easy to produce large-area films [178].

In addition, the preparation of 10 nm AlN with a *P*_r_ of 3 μC cm^−2^ using atomic layer deposition (ALD) technology has been reported [182], and with deposition carried out at 300 °C [183]. Deposition of AlScN by ALD hold promise, as it is compatible with existing CMOS processes and has the potential to be used for the fabrication of complex structures such as 3D NAND and gate-all-around FET. However, it is necessary to prepare a Sc precursor source with low evaporation temperature, and overcome problems such as oxygen defects caused by the poor vacuum level (~ 5 Pa) of ALD. Each of the aforementioned deposition techniques has its own advantages. However, it is no doubt that MS is the most favorable technology for depositing AlScN thin films from the perspectives of cost, reproducibility, adhesion of films to substrates, flexibility of deposition target materials and potential for wafer-level manufacturing.

## AlScN FeM and Its Application in IMC

Research interest in ferroelectric AlScN has been aroused due to its potential in low-power memory and neuromorphic computing. This chapter aims to present an overview of AlScN-based FeM from the perspective of commercial memory requirements, and to explore its application in IMC.

### Potential Application of AlScN in FeRAM

The current commercial DRAM mainly adopts the memory cell structure of one transistor plus one capacitor (1T1C). The capacitor is like a "cistern" responsible for storing the charge as information, and the transistor works as a "faucet" to avoid the loss of charge over time [92, 184, 185]. FeRAM shares similar structure with DRAM, except that the dielectric layer is replaced by ferroelectric material with the remanent polarization charge encoded as information [186]. Since FeRAM adopts metal–ferroelectric–metal (MFM) structure in which the ferroelectric polarization is nonvolatile, data retention is extended and the refresh operation to prevent data loss can be omitted, therefore reducing power consumption. Besides, the nanosecond or even sub-nanosecond polarization switching speed endows fast operation in FeRAM. Despite these advantages of FeRAM, several issues remain to be resolved. With the density scaling of FeRAM, it would be preferred to use ferroelectric materials of high *P*_r_ and low dielectric constant to increase the sensing margin. Besides, the reading operation of FeRAM is destructive as DRAM [187]. The charge stored in the capacitor is selectively turned on by the transistor and released to the sense amplifier to determine the storage state. After each reading, reprograming is indispensable to restore information; therefore, the capacitor should have sufficiently high endurance and moderate *E*_c_ is desirable. Because of pulse-width dependence of the switching voltage, the trade-off between operating speed and voltage necessitates an electric field twice that of the coercive field for operation. On one hand, a large *E*_c_ permits a big memory window and excellent retention behavior. On the other hand, given that operation voltage scales with the coercive field *E*_c_ and the thickness of ferroelectric film thickness, the *E*_c_ of thin AlScN films should be reduced for compatibility with CMOS logic [187]. The wurtzite-structured ferroelectrics with high *P*_r_ and low *ε* seem to be promising for FeRAM only if moderate *E*_c_ can be achieved in thin films through optimizing doping concentration and strain level [188].

Despite FeRAM based on wurtzite ferroelectrics has not been reported, MFM capacitors with ferroelectric AlScN are under intensive investigations. Wang et al*.* designed Al/Al_0.68_Sc_0.32_N/Al ferroelectric capacitor with *P*_r_ of 115 μC cm^−2^, which can achieve switching operation within 200 ns, and has no obvious fatigue behavior after 8.7 × 10^3^ cycles [161]. In the process of reducing the thickness of AlScN, Schönweger et al*.* achieved switching operation of sub-5 nm AlScN at 1 V voltage. Liu et al. unveiled that the electron emission and hopping assisted by N vacancies in the Al_0.7_Sc_0.3_N MFM capacitor dominate the leakage current in Pt/Al_0.7_Sc_0.3_N/Mo capacitor [178].

### AlScN-Based FeD and FTJ

**Fig. 11:**
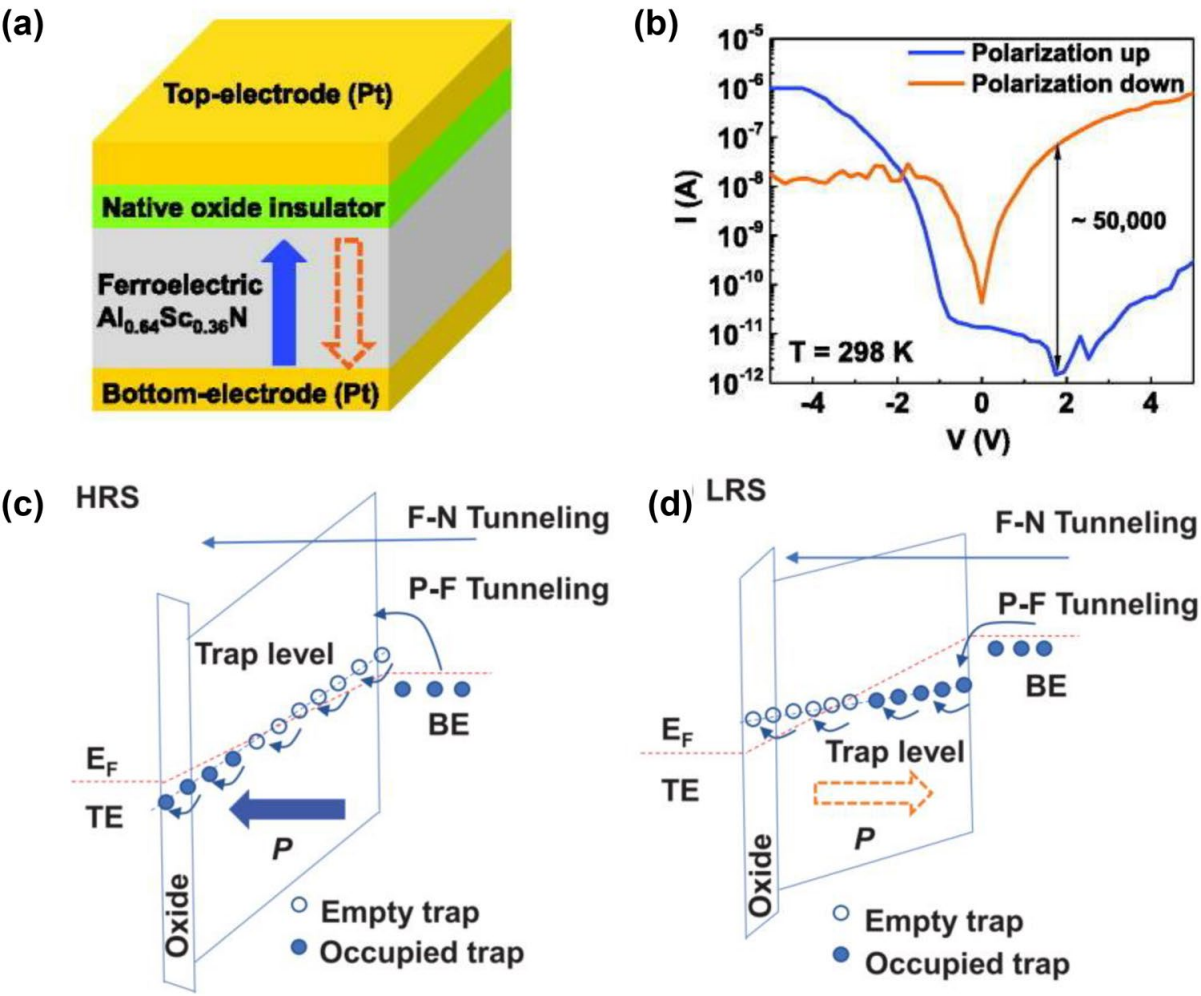
**a** Al_0.64_Sc_0.36_N-based FeD. **b** Semi-log *I*–*V* curve characteristics of the FeD. Electronic band diagrams of **c** HRS and **d** LRS in the FeD. Reproduced with permission from Ref. [160]. Copyright 2021 AIP Publishing

Although FeDs share similar MFM structure with FeRAM, the nondestructive read operation of FeDs is performed by sensing the current across the heterostructure. Taking the interfacial energy band into account, the barrier height/width modulation at the ferroelectric–electrode interface through polarization reversal was proposed for resistive switching mechanism, usually accompanied by diode-like rectifying characteristics. The polarization-dominated mechanism requires ferroelectric layer with high *P*_r_. Liu et al*.* fabricated Pt/insulator/Al_0.64_Sc_0.36_N/Pt FeD with memristor behavior, the current on/off ratio is 5 × 10^4^ (Fig. 11a, b) [160]. The leakage currents of HRS and LRS are trap-assisted conduction. The ferroelectric polarization charges modulate the band diagram, with a lower barrier height for polarization directed to electrode interface than that for polarization anti-orientated to electrode interface. For the so-called “LRS,” injected electrons jump from occupied traps to empty traps, resulting in high current. Conversely, the electron jumping rate significantly decreases (Fig. 11c, d). Asymmetric structures are usually designed at both ends of FeD to improve the switching ratio and rectification, such as metal–ferroelectric–insulator–metal (MFIM), MFS, MFIS, etc. Large rectifying ratio combined with high nonlinearity can eliminate the need for an access transistor or ovonic threshold switches in array integration. Of course, the depolarization field in the MFIM structure cannot be ignored, which requires a ferroelectric material with a suitably large *E*_c_ to offset the depolarization field. Wang et al*.* further increased the ON/OFF ratio to 10^5^ by introducing an oxide layer at the interface between the electrode and AlScN. Since the thickness of ferroelectric layer in FDs generally ranges from tens to hundreds of nanometers, the high switching voltages hinder integration with advanced CMOS nodes and the readout currents limit their miniaturization. Moreover, some experimental results have shown that the resistive switching behavior in FDs could be attributed to filament forming/rupture induced by atomic–ionic transport, which are polytropic in nature, thus suffering from small ON/OFF ratio, undesirable variations, poor retention and large cycle-to-cycle/device-to-device randomness. In some cases, both polarization-dominated mechanism and filament-dominated mechanism are known to co-dominate the resistive switching.

As the thickness of the ferroelectric layer is reduced to a few nanometers, quantum mechanical tunneling through ferroelectrics becomes possible, and the electrical conductivity is greatly enhanced for quantum mechanical tunneling through ferroelectrics and the operating voltage is decreased. These devices with a ferroelectric tunnel barrier sandwiched between two electrodes are called FTJs [189]. The height of the tunneling barrier of FTJ is determined by the state of polarization, so the domain configurations can control the tunneling conductance. Generally, ferroelectric materials with high *P*_r_ can deplete the charge at the interface of the FTJ electrode, which increases the width of the tunneling barrier and suppresses the off-state current, giving a large ON/OFF ratio [190]. Of course, by artificially designing asymmetric structures (for example, designing MFS and MFIS) to control the band offset of the tunneling barrier, this can lead to inconsistent shielding lengths at the electrode interfaces on both ends of the device, which can also increase the switching ON/OFF ratio. However, the depolarization field of the above asymmetric structures tends to be large, which requires the ferroelectric layer to have a high *E*_c_ to shield the depolarization field. Considering the requirements of the ferroelectric layers in FTJs, AlScN with large *P*_r_ and high *E*_c_ has great potential for FTJ-based products.

### AlScN-Based FeFETs

In pervasive metal–oxide–semiconductor field effect transistor (MOSFET), the gate voltage applied across the oxide insulator would accumulate or deplete carriers in semiconductor channel, realizing the essential transistor functions of switching and gain, and remaining the backbone of modern electronics. With ferroelectrics as gate insulator, FeFETs yield impressive advances. The polarization charge of traditional ferroelectrics is one or two orders higher than that of oxide dielectrics, inducing more effective modulation of channel conductance in nonvolatile manner. In addition, the continuous and reversible conductance states of FeFETs can be achieved by gradually changing the arrangement of ferroelectric domains. The multiple storage capacity is suitable for high-density data storage and neuromorphic computing. Remarkably, the two-terminal ferroelectric devices have intrinsic drawback for a single shared reading and writing path. FeFETs effectively alleviate this issue by the separation of reading (drain) and writing (gate) terminals.

**Fig. 12:**
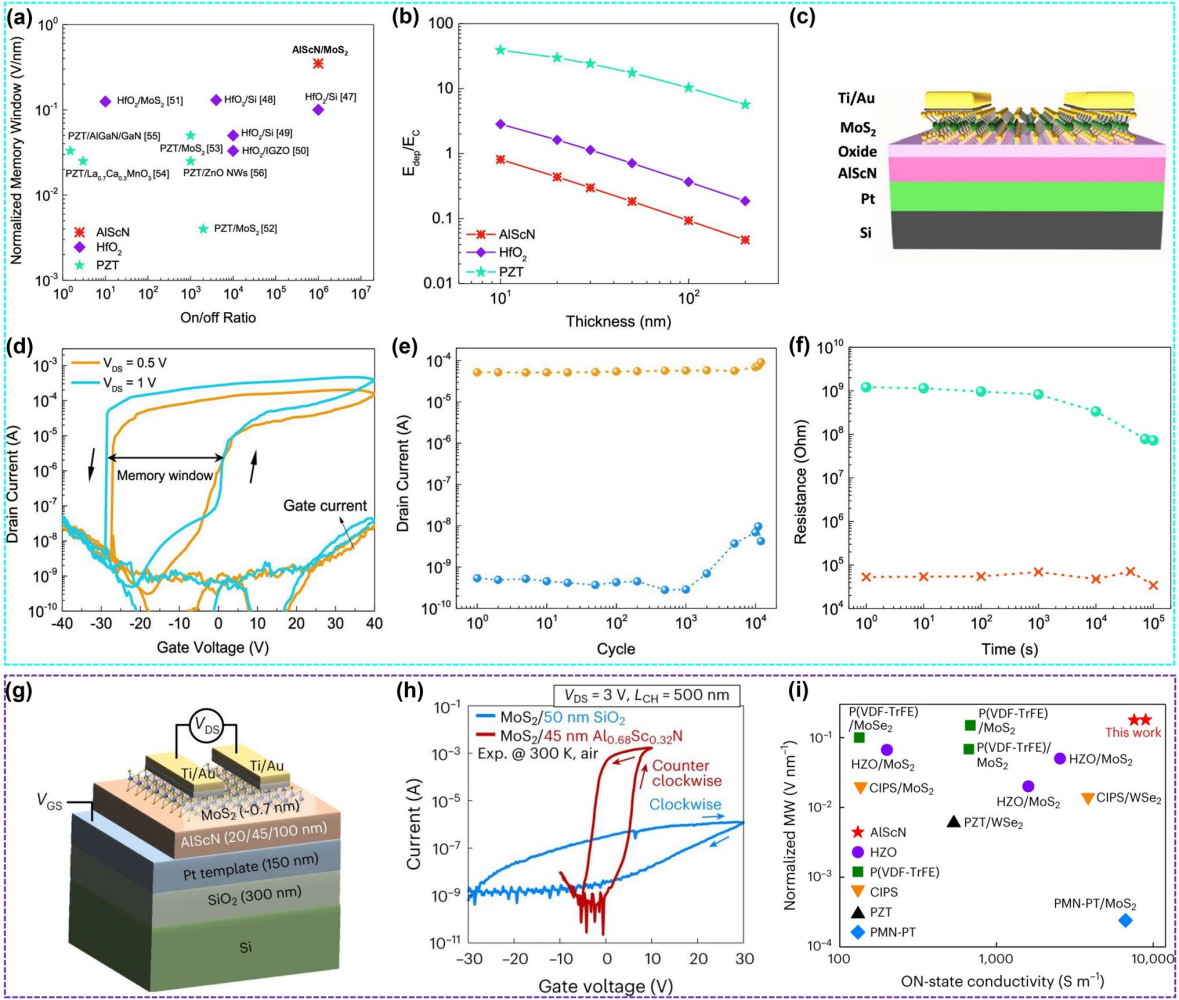
**a** ON/OFF ratio and normalized storage window of FeFETs (HZO, PZT and AlScN). **b** Simulated values of the ratio of *E*_DEP_/*E*_c_ for HZO, PZT and AlScN FeFETs. **c** Schematic of AlScN/MoS_2_ based FeFETs. **d** *I*–*V* transfer curve. **e** Endurance and **f** retention of AlScN/MoS_2_-based FeFETs. Reproduced with permission from Ref. [158]. Copyright 2021 American Chemical Society. **g** Schematic diagram of AlScN/MoS_2_ based FeFETs. **h** *I*–*V* transfer curve. **i** Comparison of normalized MW and on-state conductivity of FeFETs with different ferroelectric materials and MoS_2_ as channel. Reproduced with permission from Ref. [191]. Copyright 2023 Springer Nature

The most significant performance merits of FeFETs device are ON/OFF ratio, memory window (MW), retention and endurance. The AlScN films with high *P*_r_ of ~ 100 µC cm^−2^ support larger carrier density modulation of approximately 10^14^ cm^−2^ than SiO_2_ gate dielectric of only about 10^13^ cm^−2^, achieving high ON/OFF ratio of 10^6^ (Fig. 12a). It should be noted that depolarization field (*E*_DEP_) can also be increased if *P*_r_ is too high, resulting in poor retention. The high *E*_c_ of AlScN makes *E*_DEP_/*E*_c_ low enough, and thus good retention can be obtained (Fig. 12b). Both MW and operating voltage are proportional to *E*_c_ and thickness of ferroelectric films. Large MW facilitated by high *E*_c_ is essential to correctly distinguish the stored states. However, the high *E*_c_ causes high driving power value and a typical endurance of < 10^5^ cycles. Based on the above analysis, to achieve better comprehensive performance, the *E*_c_ of AlScN film should be modified by changing the Sc concentration, strain engineering and/or superlattice/multilayer construction. It is worth mentioning that the low permittivity of AlScN is favorable to reduce the electric field in the interfacial layer and improve endurance cycling in MFIS-structured FeFETs.

During the preparation process of FeFETs, there may be defects in the ferroelectric–semiconductor interface, resulting in interface reaction or diffusion, which weakens the quality of ferroelectric layer, and affects the modulation efficiency of FeFETs. Therefore, the ferroelectric–semiconductor interface quality is the key factor determining the performance of FeFETs. As mentioned in Sect. 3.4, most reported Al_1−*x*_Sc_*x*_N films have been on highly oriented fcc or bcc metals or TiN, which provide a hexagonal template for growing [001]-direction wurtzite ferroelectrics. There are various wide-bandgap semiconductors such as GaN and SiC that are some degree of lattice matching with Al_1−*x*_Sc_*x*_N films, but their integration efforts are mainly focused on high-frequency and high-electron mobility devices. 2D materials provide a solution due to free from dangling bonds, low short-channel effects and ability to be transferred onto different substrates. Liu et al*.* demonstrated high-performance FeFET with AlScN as dielectric and 2D MoS_2_ as channel (Fig. 12c–f), exhibiting a record normalized memory window (30–40 V) and ON/OFF ratio (10^6^), simultaneously with good retention (10^5^ s), cycling endurance (> 10^4^) and CMOS BEOL compatible processing temperatures (approximately 350 °C) [158]. In this work, MoS_2_ flake was mechanically exfoliated from its bulk crystal, a poor option in terms of large-scale applications. Kim et al*.* adopted CVD and MOCVD methods to prepare large-area MoS_2_ films with accurate thickness control (Fig. 12g–i) [191]. A large array of high-performance and scalable FeFETs was demonstrated. Impressively, thanks to the high *P*_r_ value of AlScN, the FeFETs hold a large MW of ~ 8 V and an ON/OFF ratio greater than 10^6^. The FeFETs display stable retention up to 10 years by extension, and endurance greater than 10^4^ cycles. Through voltage-tunable partial switching of ferroelectric domains, 4-bit pulse-programmable memory function and 7-bit operation as artificial synapses are explored. All these merits of MoS_2_/AlScN FeFET arrays pave the way toward the ferroelectric memory with silicon CMOS logic.

The unavoidable depolarization field in FETs, the low *E*_c_ of conventional ferroelectrics and the unsatisfactory ON/OFF ratio hinder the commercial application of FeFETs [192]. Fortunately, ferroelectric AlScN has large *E*_c_ to shield the depolarization field and large *P*_r_ to modulate the channel conductance withe large ON/OFF ratio, and meets the requirements of CMOS compatibility. Meanwhile, compared with other ferroelectric materials, AlScN has lower permittivity, which increases the voltage loading in the ferroelectric layer. The fatigue characteristics of AlScN FeFETs are already comparable to flash memory, and the read and write speeds are faster than flash memory. Note that flash memory has extremely high integration density and reliability, from the potential commercial view, 3D integration of AlScN FeM needs to be explored.

### AlScN-Based FeM for IMC

**Fig. 13:**
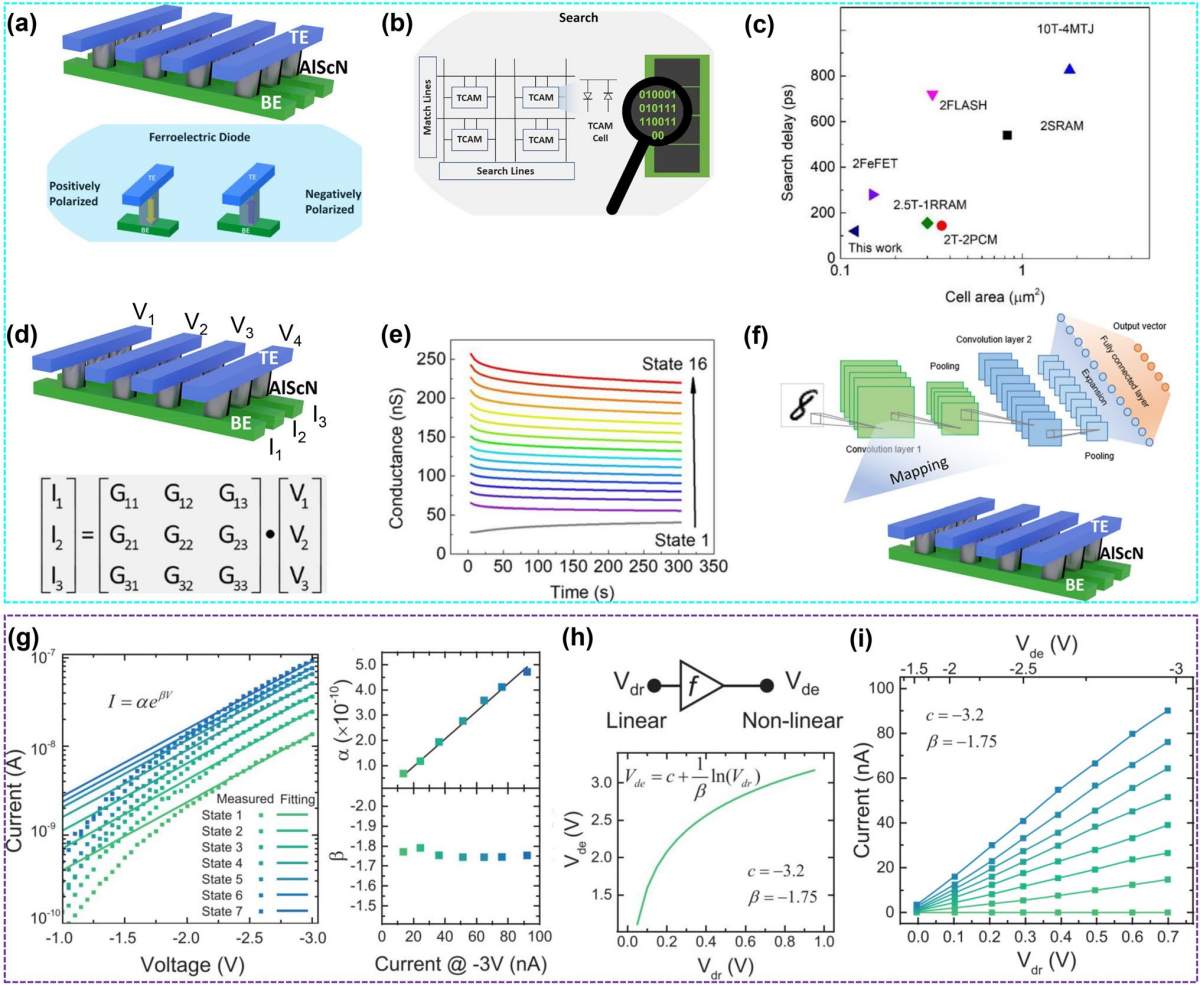
**a** and **b** Two-FeD TCAM cell for search operation. **c** Benchmark comparison chart of lateral footprint of various TCAM cells vs. search delay. **d** AlScN FD crossbar array implementation of VMM. **e** Retention properties of 16 conductance states. **f** Topology diagram of convolutional neural network. Reproduced with permission from Ref. [163]. Copyright 2022 American Chemical Society. **g** Fitting of the *I*–*V* curves. **h** and **i** Schematic of a logarithmic driver which maps linear *V*_dr_ to nonlinear *V*_de_ for AlScN memristors. Reproduced with permission from Ref. [135]. Copyright 2022 Wiley-VCH

As mentioned in Sect. 1, IMC is a solution to solve the memory wall problem [193]. The memory and processor in a von Neumann computer system are separated. The overhead of frequent processor access to memory forms a memory wall [194]. The basic idea of IMC is to improve the computing power of memory through circuit innovation, thereby reducing the frequency of processor access to memory [195]. AI computing requires several basic operations, such as on-chip storage, on-chip parallel search and on-chip VMM, which poses challenges to the reconfigurability and operational flexibility of IMC architectures. On-chip storage is a basic function of all NVM. On-chip parallel search operations require multiple devices to work together and these devices can store states. For example, the operation of ternary content addressable memory (TCAM) requires 16 transistors in CMOS circuits. However, the operation of TCAM based on RRAM also requires 2T2R to complete. Recently, Liu et al. demonstrated that TCAM operation using two AlScN-FeD with opposite polarity as the basic unit (0.12 μm^2^/cell) can support three states: "match," "mismatch" and "don't care," with search latency within 100 ps (Fig. 13a–c).

On-chip VMM requires multiple states (such as conductance states) of devices, which is the foundation for neuromorphic computing. Liu et al. developed an AlScN-based FeD crossbar array for IMC, and realized different conductance states by adjusting the polarization states of AlScN-based FeD. This allows the VMM of the input voltage of a word line with the conductance matrix to read the accumulated current on the FeD bit line (Fig. 13d). The FeD unit can be modulated into 16 different conductance states with excellent retention characteristics (Fig. 13e). The conductance is normalized as a weight and stored in the FeD array for VMM. The accuracy of digital recognition (95.5%) for FeD in situ training is only 2% lower than that of software simulation. (Fig. 13f) [163]. For crossbar arrays with good linearity, matrix operations can be directly performed, while nonlinear FeMs require additional fitting of the nonlinear output signal with the linear input pulse signal to obtain linear conductance. Wang et al*.* designed an ultra-thin ScAlN (5 nm)/GaN heterojunction memristor with good retention (> 10^5^ s) and cycling endurance (> 10^4^), and can be programmed to eight stable conduction states (Fig. 13g). The *I*–*V* characteristics are fitted using an exponential function, and constant *β* and linear *α* values are observed at the conductance level. A mapping function is used to scale the nonlinear device voltage (*V*_de_) to a linear drive voltage (*V*_dr_). The mapping results demonstrate the realization of linear conductance (Fig. 13h, i). The ScAlN ferroelectric memristor has achieved convolutional processing operations such as main extraction, edge extraction and sharpen, and the accuracy of digital recognition has reached 92.9% [196]. It should be noted here that this memristor was grown on GaN/sapphire substrate, but the n-GaN layer can be replaced with other metals such as Al or Mo, which can be prepared on Si through CMOS compatible processes. Therefore, the memristor and peripheral circuits can be manufactured using BEOL processes. In the latest report of this group, the GaN/ScAlN/Mo/Sc_2_O_3_/Si heterostructure has been used to simulate the spike-time-dependent plasticity of biological synapses, demonstrating the prospects of ScAlN-based heterostructures for integration with CMOS processes and neuromorphic computing [197].

It is important to emphasize that accurate weights and weight updating are essential for neuromorphic device that is capable of multi-bit operations. When manufacturing ferroelectric-based neuromorphic devices, it is necessary to understand the relationship between conductance and domain states. Thus, the domain dynamics play an important role for realizing these synaptic characteristics of weight updating.

## Conclusions and Outlook

Ferroelectric AlScN has advantages such as CMOS back-end of line compatibility, sustainable miniaturization, intrinsic and stable-phase ferroelectricity, which has spurred researchers to extensively explore its physical properties. This review provides a comprehensive summary of AlScN-based ferroelectrics, covering aspects such as the ferroelectric mechanism and domain dynamics, FeMs and their IMC applications. Despite these advancements, the properties of AlScN still require further refinement and development to realize their full potential in future commercial applications.

### The Challenges at the Material Level

The doping of the group III elements can induce stress and reduce the free energy barrier in III-nitrides, leading to polarization reversal of III-nitrides before hard breakdown and accompanied by huge *P*_r_. It is imperative to systematically analyze the influence of factors such as vacancies, temperature, strain, doping concentration, impurities, uneven domains and surface adsorption on ferroelectric properties of AlScN. Currently, in situ PFM combined with pulse testing has confirmed the domain inversion dynamics in AlScN, and the presence of AlScN ferroelectric domain walls has been confirmed with STEM and other methods. However, further investigation using high-precision in situ X-ray diffraction (XRD) and in situ STEM characterization methods is necessary to unravel the origin of AlScN's ferroelectricity and the temperature dependence of ferroelectric properties.

### Demand for AlScN in Commercial Memory

Based on the actual demand of FeM mentioned in Sect. 4, huge *E*_c_ is a double-edged sword. The MW of FeFETs is mainly determined by the coercive voltage (*V*_c_) of the ferroelectric layer, proportional to the thickness of the ferroelectric film and the *E*_c_. The miniaturization of devices necessitates a reduction in the gate thickness. Therefore, in order to maintain the required MW, the ferroelectric gate material in FeFETs devices needs to possess large *E*_c_. Moreover, the incomplete shielding by semiconductor channels in FeFETs introduces large depolarization field, resulting in poor data retention time. High *E*_c_ is beneficial for improving the retention characteristics of the device. However, the extremely large *E*_c_ requires higher operating voltages and limits the endurance ability of FeRAM. The cycling at high voltages can lead to performance degradation. Consequently, moderate *E*_c_ is deemed optimal for achieving balanced performance in ferroelectric applications. The huge *E*_c_ of AlScN can be reduced by adjusting the concentration of cations such as the incorporated Ga, B and Sc. Changing the substrate or employing rapid annealing to increase in-plane tensile stress can also decrease *E*_c_ [198]. Additionally, the adoption of ultra-thin film is necessary to lower the required voltage level. Should the operating voltage of AlScN be tailored to be compatible with the silicon process, it would unlock boundless potential of AlScN in advanced electronics manufacturing.

The remanent polarization *P*_r_ is another critical parameter of ferroelectric materials. While large *P*_r_ offers advantages, it also poses challenges. The substantial *P*_r_ exhibited by AlScN expands the range of remanent polarization options and enables the generation of strong local electric fields for effective electrostatic doping [12, 26]. For instance, adjusting the amplitude of the applied voltage can yield numerous inner hysteresis loops with varying *P*_r_ values. However, in FeFETs, large *P*_r_ can result in significant depolarization field. Therefore, in FeFETs applications, a suitable *P*_r_ is preferred to balance performance and stability.

When the applied electric field approaches close to *E*_c_, AlScN experiences significant leakage current, resulting in poor cyclic durability. This issue is usually attributed to nitrogen defects, dislocations and uneven domains. A high vacuum deposition system is required to grow thin films in an environment with high nitrogen content to inhibit the formation of nitrogen vacancies. Additionally, fit substrates which can not only provide epitaxial templates to suppress dislocations and uneven domains but also induce appropriate strain to flatten polarization energy profile are indispensable. Furthermore, it is also important to systematically study wake-up effect, imprinting effect, fatigue failure mechanism and cyclic durability mechanism that need to be addressed for the storage application of wurtzite ferroelectric thin films.

In order to meet the demand for AlScN in commercial chips, it is imperative to focus on cost reduction while maintaining the quality of AlScN wafers. Additionally, meeting the requirements of the deep hole filling process is essential. As discussed in Sect. 3.4, the ALD process of AlScN can be employed for deep hole filling of intricate structures, but the defects such as lattice oxygen caused by poor vacuum condition in ALD remain significant concerns. Furthermore, leveraging the performance advantages of wurtzite ferroelectric materials opens avenues for designing novel memory structures and chip architectures.

### Demand for FeM Chips for IMC

In performing VMM computation, the uniformity across devices and cycles is paramount. This underscores the critical need for devices that exhibit consistent performance and high durability, necessitating a deep understanding of the intricate connections between polarization states, domain dynamics, and resistance within the device. At present, AlScN-based devices remain relatively rudimentary, often existing in simplistic configurations as single units. To advance toward IMC, there is a pressing demand for high-quality, large-scale fabrication of device arrays. For FTJ and FeD, achieving sufficiently compact unit sizes and enabling multilevel states are crucial for optimal performance. In the case of FeFETs, adapting NAND-like structures presents a viable approach to enhance storage density.

**Fig. 14:**
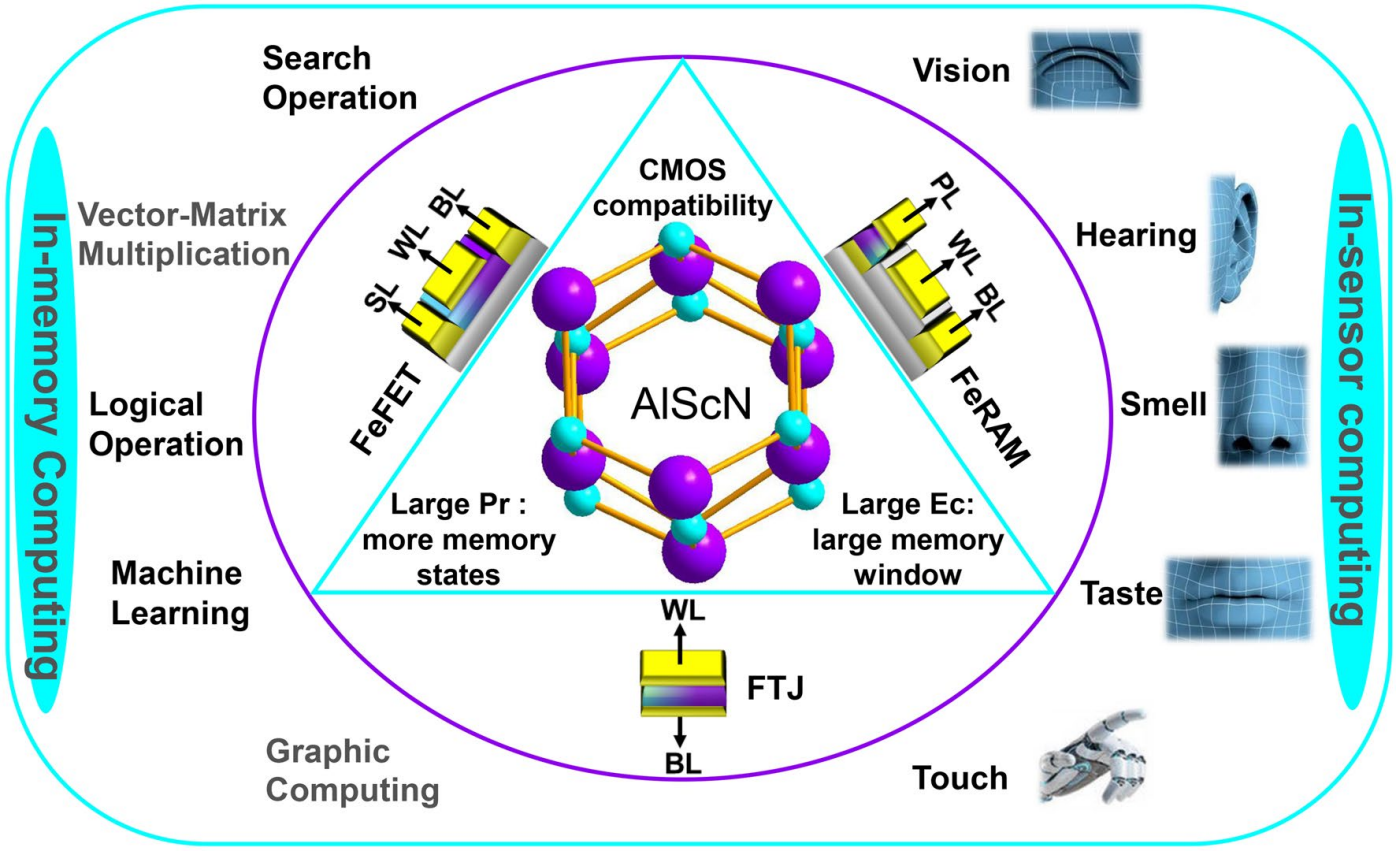
Advantages of ferroelectric AlScN in NVM and its future application in the field of IMC and in-sensor computing

In summary, ferroelectric AlScN demonstrates excellent ferroelectricity and has broad application prospects in the field of ferroelectric NVM. While addressing the aforementioned challenges, it is crucial to focus on adjusting the electrical properties of ferroelectric AlScN and exploring an integrated process compatible with both front-end and back-end technologies. Of course, it is essential to expand the application of AlScN in IMC (such as search operation, vector–matrix multiplication, logical operation, machine learning and graphic computing) and in-sensor computing (such as artificial vision, hearing, touch, smell and taste sensors) (Fig. 14). Despite the potential time required for commercialization, it is anticipated that the outstanding performance of AlScN will lead to widespread applications once its films and devices achieve critical mass.
